# Utility of Human *In Vitro* Data in Risk Assessments of Influenza A Virus Using the Ferret Model

**DOI:** 10.1128/jvi.01536-22

**Published:** 2023-01-05

**Authors:** Hannah M. Creager, Troy J. Kieran, Hui Zeng, Xiangjie Sun, Joanna A. Pulit-Penaloza, Katie E. Holmes, Anders F. Johnson, Terrence M. Tumpey, Taronna R. Maines, Catherine A. A. Beauchemin, Jessica A. Belser

**Affiliations:** a Influenza Division, Centers for Disease Control and Prevention, Atlanta, Georgia, USA; b Emory University, Atlanta, Georgia, USA; c Department of Physics, Ryerson University, Toronto, Canada; d Interdisciplinary Theoretical and Mathematical Sciences (iTHEMS) at RIKEN, Wako, Japan; Emory University School of Medicine

**Keywords:** epithelial cells, ferret, influenza, risk assessment, virus replication

## Abstract

As influenza A viruses (IAV) continue to cross species barriers and cause human infection, the establishment of risk assessment rubrics has improved pandemic preparedness efforts. *In vivo* pathogenicity and transmissibility evaluations in the ferret model represent a critical component of this work. As the relative contribution of *in vitro* experimentation to these rubrics has not been closely examined, we sought to evaluate to what extent viral titer measurements over the course of *in vitro* infections are predictive or correlates of nasal wash and tissue measurements for IAV infections *in vivo*. We compiled data from ferrets inoculated with an extensive panel of over 50 human and zoonotic IAV (inclusive of swine-origin and high- and low-pathogenicity avian influenza viruses associated with human infection) under a consistent protocol, with all viruses concurrently tested in a human bronchial epithelial cell line (Calu-3). Viral titers in ferret nasal wash specimens and nasal turbinate tissue correlated positively with peak titer in Calu-3 cells, whereas additional phenotypic and molecular determinants of influenza virus virulence and transmissibility in ferrets varied in their association with *in vitro* viral titer measurements. Mathematical modeling was used to estimate more generalizable key replication kinetic parameters from raw *in vitro* viral titers, revealing commonalities between viral infection progression *in vivo* and *in vitro*. Meta-analyses inclusive of IAV that display a diverse range of phenotypes in ferrets, interpreted with mathematical modeling of viral kinetic parameters, can provide critical information supporting a more rigorous and appropriate contextualization of *in vitro* experiments toward pandemic preparedness.

**IMPORTANCE** Both *in vitro* and *in vivo* models are employed for assessing the pandemic potential of novel and emerging influenza A viruses in laboratory settings, but systematic examinations of how well viral titer measurements obtained *in vitro* align with results from *in vivo* experimentation are not frequently performed. We show that certain viral titer measurements following infection of a human bronchial epithelial cell line are positively correlated with viral titers in specimens collected from virus-inoculated ferrets and employ mathematical modeling to identify commonalities between viral infection progression between both models. These analyses provide a necessary first step in enhanced interpretation and incorporation of *in vitro*-derived data in risk assessment activities and highlight the utility of employing mathematical modeling approaches to more closely examine features of virus replication not identifiable by experimental studies alone.

## INTRODUCTION

Influenza A viruses (IAVs) are capable of overcoming species barriers to cause sporadic cases of human infection. While most zoonotic-to-human transmission events are self-limiting, there remains the potential during this process for the acquisition of features associated with enhanced mammalian adaptation, which could result in the emergence of a pandemic virus. Risk assessment rubrics (notably the Influenza Risk Assessment Tool [IRAT] and the World Health Organization Tool for Influenza Pandemic Risk Assessment [TIPRA]) have been established to consider virus, host, ecological, and environmental properties to facilitate the assessment of the public health risk posed by novel and emerging influenza viruses (1, 2). These models are informed by *in vivo* pathogenicity and transmissibility data generated in laboratory models, specifically the ferret. *In vitro* experimentation is frequently employed concurrently with *in vivo* assessments (and in the case of TIPRA, is formally considered) to provide supporting information to *in vivo* data. These *in vitro* assessments are typically conducted in cultured human respiratory epithelial cell lines but have expanded in recent years to include primary cells, tissue explants, tissue constructs, and organoid cultures (3, 4).

To assess mammalian pathogenicity of novel and emerging IAVs associated with human infection or exposure, serologically naive ferrets are typically inoculated intranasally with a high dose of virus (10^5^ to 10^7^ infectious units) and observed daily for clinical signs of infection, with specimens collected during the acute phase of infection from the upper respiratory tract. However, the variability of experimental conditions between laboratories performing risk assessment studies represents a challenge for contextualizing results in the field (5). Even within controlled laboratory environments, the wide heterogeneity of influenza virus subtypes associated with human infection can lead to complications for their comparative study (6). Study-to-study comparisons of viral growth kinetics in cells derived from the human respiratory tract are often similarly hindered due to the use of different cell types between studies and the limited availability of cultures that are not commercially available (4).

While previous studies have examined the association of *in vivo* ferret data as they pertain to human pathogenicity and transmissibility (7, 8), there remains a need to understand the strengths and limitations of *in vitro* characterizations performed at a fixed multiplicity of infection (MOI) to mammalian risk assessment activities. Zoonotic IAVs typically replicate to high and sustained titer at both 37°C and 40°C (the temperature of the avian enteric tract), whereas a shorter time to reach comparable titer metrics at 33°C (the temperature of the human nasal passages) could be indicative of mammalian adaptation (9, 10), although this has not been systematically examined. *In vitro* results are typically presented as a capacity to replicate to high titer, to indicate relative levels of host adaptation, and as a potential to replicate efficiently in human respiratory tract tissues, often contextualized with additional parameters such as elicitation of host responses (11, 12). As *in vitro* models become more advanced, so must our understanding of the capacity of these models to recapitulate *in vivo* environments. In this vein, mathematical models (MMs) of replication dynamics and their use to identify differential growth properties between human and zoonotic influenza viruses in relevant human cell types represent a sophisticated approach to improving our knowledge of host adaptation processes (13). An *in vitro* MM of IAVs with pandemic potential distills viral titer data into key parameters characterizing their replicative fitness, providing valuable information for both laboratory and epidemiological applications (14).

Here, we perform an exploratory analysis comparing IAV titer measurements and some MM-derived quantities *in vitro* and *in vivo*, employing data obtained in the human bronchial epithelial cell line Calu-3 with paired data obtained from virus-inoculated ferrets. Analyses were conducted using titers generated by viral titration during standard replication kinetics experiments *in vitro*, and MM parameters were estimated from the *in vitro* infections for each virus strain. The inclusion of contemporary human and zoonotic IAVs that exhibit differential mammalian pathogenicity permits the investigation and identification of features generated from *in vitro* assessments that are most indicative of *in vivo* mammalian replication fitness. Collectively, this study provides a necessary first step toward improved quantitative interpretation of *in vitro* data generated for risk assessment purposes.

## RESULTS

### Control of parameters for *in vivo* and *in vitro* assessments.

Virus pathogenicity and titration data were aggregated from ferrets inoculated with a wide range of 52 contemporary human and zoonotic IAVs (isolated from 1999 to 2018), employing a uniform experimental protocol for all experiments to minimize laboratory- or protocol-based confounders (Table 1) (5). Nasal wash (NW) specimens (inclusive of virus replication in multiple locations in the ferret upper respiratory tract [15]) were collected from all virus-inoculated ferrets; mean peak NW titer, reflecting the maximum viral titer days 1 to 5 postinoculation (p.i.) from *n* ≥ 3 ferrets, was determined for each virus (reported in Table S1 in the supplemental material). With few exceptions, mean viral titers from respiratory tract tissues collected on day 3 p.i. (nasal turbinates, trachea, and lung) were included in analyses as specified in Materials and Methods.

**TABLE 1:** Influenza A viruses evaluated in ferrets and Calu-3 cells

Virus name	Subtype^*a*^	Description^*b*^	Titration units^*c*^	PB2 627^*d*^	33°C^*e*^	References^*f*^
A/Brisbane/59/2007	H1N1	Human seasonal	PFU	K	X	11, 43, 44
A/California/4/2009	H1N1pdm09	Human pandemic	PFU	E	X	11, 43, 45
A/Mexico/4482/2009	H1N1pdm09	Human pandemic	PFU	E	X	11, 43
A/Texas/15/2009	H1N1pdm09	Human pandemic	PFU	E	X	11, 43
A/Netherlands/1132/2009	H1N1pdm09	Human pandemic	PFU	E	X	46, this study
A/Ohio/2/2007	H1N1v	Variant swine	PFU	E	X	11, 47, 48
A/Texas/14/2008	H1N1v	Variant swine	PFU	E	X	11, 47, 48
A/Iowa/39/2015	H1N1v	Variant swine	PFU	E	X	45, 48
A/Ohio/9/2015	H1N1v	Variant swine	PFU	E	X	45, 48
A/Hunan/42443/2015	H1N1v	Variant swine	PFU	E	X	49, this study
A/Minnesota/19/2011	H1N2v	Variant swine	PFU	E	X	48
A/Minnesota/45/2016	H1N2v	Variant swine	PFU	E	X	48
A/Wisconsin/71/2016	H1N2v	Variant swine	PFU	E	X	48
A/Panama/2007/1999	H3N2	Human seasonal	PFU, EID_50_	K	X	50, 51, this study
A/Perth/16/2009	H3N2	Human seasonal	PFU	K		52, 53
A/canine/Illinois/12191/2015	H3N2 canine	Canine isolate	EID_50_	E	X	54
A/Kansas/13/2009	H3N2v	Variant swine	PFU	E		51, 53
A/Minnesota/11/2010	H3N2v	Variant swine	PFU	E		51, 53
A/Pennsylvania/14/2010	H3N2v	Variant swine	PFU	E		51, 53
A/Indiana/8/2011	H3N2v	Variant swine	PFU	E		51 – 53
A/Iowa/8/2011	H3N2v	Variant swine	PFU	E		51
A/Ohio/13/2012	H3N2v	Variant swine	PFU	E		51
A/Michigan/39/2015	H3N2v	Variant swine	PFU	E		51
A/Ohio/27/2016	H3N2v	Variant swine	PFU	E		51
A/Thailand/16/2004	H5N1	HPAI (Eurasian)	EID_50_	K	X	41, this study
A/Vietnam/1203/2004	H5N1	HPAI (Eurasian)	EID_50_	K	X	41, this study
A/Bangladesh/5487/2011	H5N1	HPAI (Eurasian)	EID_50_	K	X	55, this study
A/duck/Vietnam/NCVD-672/2011	H5N1	HPAI (Eurasian)	EID_50_	E	X	55, this study
A/chicken/Texas/18-007912-2/2018	H7N1	LPAI (N. American)	EID_50_	E	X	56
A/turkey/Virginia/4529/2002	H7N2	LPAI (N. American)	EID_50_	E	X	57 – 59
A/New York/107/2003	H7N2	LPAI (N. American)	EID_50_	E	X	57 – 59
A/New York/108/2016	H7N2	LPAI (N. American)	EID_50_	E	X	59
A/Canada/504/2004	H7N3	HPAI (N. American)	EID_50_	E		39, 46
A/Mexico/InDRE7218/2012	H7N3	HPAI (N. American)	EID_50_	E	X	56, 60
A/turkey/California/18-031151-4/2018	H7N3	LPAI (N. American)	EID_50_	E	X	56
A/Netherlands/219/2003	H7N7	HPAI (Eurasian)	EID_50_	K	X	56 – 58
A/Netherlands/230/2003	H7N7	HPAI (Eurasian)	EID_50_	E	X	56 – 58
A/Italy/3/2013	H7N7	HPAI (Eurasian)	EID_50_	E	X	56, 61
A/turkey/Indiana/1403/2016	H7N8	HPAI (N. American)	EID_50_	E		62
A/turkey/Indiana/1573-2/2016	H7N8	LPAI (N. American)	EID_50_	E		62
A/goose/Nebraska/17096-1/2011	H7N9	LPAI (N. American)	EID_50_	E	X	56, 60, 62, 63
A/chicken/Tennessee/17-007147-2/2017	H7N9	HPAI (N. American)	EID_50_	E		63
A/chicken/Tennessee/17-007431-3/2017	H7N9	LPAI (N. American)	EID_50_	E		63
A/shoveler/Egypt/00215-NAMRU3/2007	H7N9	LPAI (Eurasian)	PFU	E	X	64
A/Anhui/1/2013	H7N9 (1)	LPAI (Eurasian)	PFU	K	X	64
A/Shanghai/1/2013	H7N9 (1)	LPAI (Eurasian)	PFU	K	X	64
A/Taiwan/1/2013	H7N9 (1)	LPAI (Eurasian)	PFU	K	X	65
A/Hong Kong/5942/2013	H7N9 (2)	LPAI (Eurasian)	PFU	K	X	65
A/British Columbia/1/2015	H7N9 (3)	LPAI (Eurasian)	PFU	K	X	65
A/Hong Kong/4553/2016	H7N9 (5)	LPAI (Eurasian)	EID_50_	K	X	66, this study
A/Guangdong/17SF003/2016	H7N9 (5)	HPAI (Eurasian)	EID_50_	E		66
A/Taiwan/1/2017	H7N9 (5)	HPAI (Eurasian)	EID_50_	K		66

a Epidemiological wave from which H7N9 viruses were isolated from humans is indicated in parentheses. v, variant virus.

b Variant swine describes human isolates of swine origin. HPAI, highly pathogenic avian influenza virus; LPAI, low pathogenic avian influenza virus. Virus lineage for avian influenza viruses is specified in parentheses.

c The method of titration for detection of infectious virus. EID_50_, 50% egg infectious dose; PFU, PFU in London-line Madin Darby Canine Kidney (MDCK) cells. For analyses split between egg and cell titration matrix, Panama/99 virus is represented in both; for combined analyses, PFU data are included.

d Amino acid at PB2 position 627. K, lysine; E, glutamic acid.

e Viruses for which paired Calu-3 data were generated at both 37°C and 33°C are indicated.

f All ferret data have been published previously. Calu-3 data have either been published previously or generated for this study as indicated.

All viruses were evaluated in the human tracheal-bronchial epithelial cell line Calu-3, one of many *in vitro* models employed to support risk assessment work (3). Calu-3 cells were grown to confluence in either 12-mm or 24-mm Transwell inserts at a liquid-liquid interface, and standard replication kinetics evaluations were conducted at 37°C using a fixed MOI of 0.01 infectious units/cell. Comparable viral replication kinetics and mean peak titers were observed following infection with a diverse range of IAVs independent of well size (Fig. S1), permitting pooled analysis of these data. Peak viral titers (24 to 72 h p.i.) from Calu-3 cells infected with the same virus were found to be comparable between replicates from multiple independent experiments, in contrast with mean titers collected at specific time points that possessed the potential for greater variability between repeat experimentation (Table S1; Fig. S2).

Quantification of infectious virus load from samples collected *in vitro* or *in vivo* was determined by titration in either embryonated chicken eggs or Madin-Darby Canine Kidney (MDCK) cells to determine a 50% egg infectious dose (EID_50_) or PFU titer, respectively. The panel of viruses employed in this study included both titration methods, with a general bias toward cell titration for human H1 and H3 subtype viruses, and egg titration for avian H5 and H7 subtype viruses (Table 1), due to strain-specific viral replication capacities in each titration matrix. Because the relationship between EID_50_ and PFU titers can vary between viruses in a strain-specific manner (Fig. S3), only viruses for which the same matrix was uniformly employed for both *in vivo* and *in vitro* sample titration were included in this study. While the majority of analyses were performed on the entire data set, for certain comparisons (notably those that compared viral titer data with nontiter measures generated from mathematical modeling), independent analyses were performed for each titration matrix.

Using this information, we conducted statistical and other predictive analyses to examine possible correlations and associations between Calu-3 and ferret viral titer data. Pearson correlation coefficients, denoted by *r*, are used throughout to quantify the extent to which two measures are correlated; correlations for which zero is excluded from the 95% confidence interval (95% CI) of the estimated *r* value were of primary interest. Because multiple comparisons were performed, the *p* values computed herein do not correspond to an absolute measure of the true significance of any one comparison. Instead, we report a quantity we call the ranking statistic (RS-*p*), which is used only to rank each correlation from most to least significant but is not to be interpreted in absolute terms. Throughout this study, viral titer is presented as log_10_ titer, and all calculations were performed with the log_10_ of the measured virus titer and not the virus titer itself.

### Correlation between ferret and Calu-3 viral titers postinfection.

**FIG 1:**
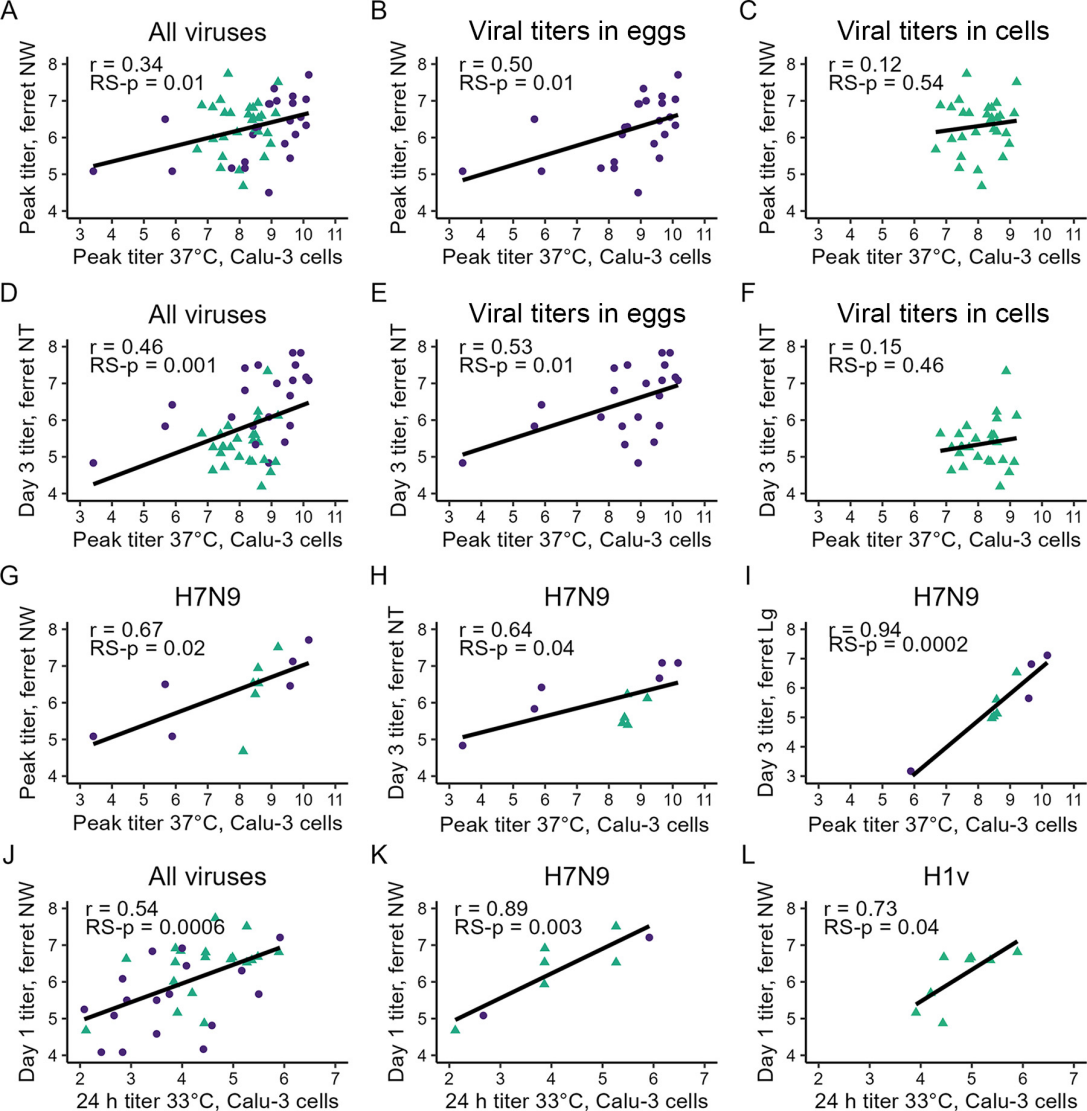
Correlations between ferret nasal wash, ferret nasal turbinate, ferret lung, and Calu-3 viral titers following IAV infection. Best-fit line determined by least-squares regression; *r* values are from Pearson correlation tests (see Table S2 in the supplemental material). Viral titers correspond to the log_10_ of the viral titers, measured in EID_50_/mL (NW, NT) or EID_50_/g (Lg) (viral titration in eggs, circles) or PFU/mL (NW, NT) or PFU/g (Lg) (viral titration in cells, triangles); sample titration matrix in mixed data sets is specified in Table 1. (A to C) Peak ferret NW versus peak Calu-3 titer (37°C) for all viruses in data set (A), the viral titers determined in eggs only (B), or viral titers determined in cells only (C). (D to F) Day 3 p.i. NT titer versus peak Calu-3 titer (37°C) for all viruses in data set (D), the viral titers determined in eggs only (E), or viral titers determined in cells only (F). (G to I) Among H7N9 subtype viruses only, peak ferret NW versus peak Calu-3 titer (37°C) (G), day 3 p.i. NT titer versus peak Calu-3 titer (37°C) (H), and day 3 p.i. Lg titer versus peak Calu-3 titer (37°C) (I). (H to L) Day 1 p.i. ferret NW versus 24-h Calu-3 titer (33°C) for all viruses in data set (J), H7N9 subtype viruses only (K), or H1v (H1N1v and H1N2v) subtype viruses only (L).

We first examined to what extent Calu-3 viral titers were predictive of viral load measured in ferret NW specimens. When employing the entire data set (all viruses listed in Table 1), peak ferret NW titers were positively correlated (*r* = 0.34 with 95% CI [0.08, 0.56], RS-*P* = 0.01) with peak Calu-3 titers from infections conducted at 37°C (Fig. 1A). When viruses were separated by titration matrix, positive Pearson correlation coefficients were higher for the viral titer determined in eggs than in cells; similar results were obtained when analyses were stratified by host origin rather than titration matrix (with higher coefficients among viruses of avian origin) (Fig. 1B and C; Table S2A). Comparable trends following data stratification were maintained between peak Calu-3 titers at 37°C and NW specimens collected on individual days p.i. (Fig. S4; Table S2A). Correlation coefficients were generally higher when analyses were limited to groups of genetically related viruses (e.g., H7N9 subtype viruses only [Fig. 1G; Table S2A]). Interestingly, compared to peak titer measurements, assessments of viral titer 24 h post *in vivo* inoculation (day 1 p.i. NW) and *in vitro* infection (24-h Calu-3 titer cultured at 37°C) were more significant both for the entire data set (*r* = 0.44, [0.19, 0.63], RS-*P* = 0.001, Fig. S4) or when restricted to H7N9 subtype viruses (*r* = 0.96 [0.85, 0.99], RS-*P* = 1e-6, Table S2A).

Similar to peak NW titer, day 3 p.i. nasal turbinate (NT) titers (all samples were sampled on day 3 p.i.) were positively correlated (*r* = 0.46, [0.20, 0.66], RS-*p* = 0.001) with peak Calu-3 titers for infections conducted at 37°C, with higher positive Pearson correlation coefficients for viruses titrated in eggs or of avian origin, or when restricted to H7N9 subtype viruses (Fig. 1D to F and H; Table S2B). In contrast to NT titers, Pearson correlation coefficients were generally lower between trachea (Tr) or lung (Lg) titers and peak Calu-3 titers cultured at 37°C when employing either the full data set or when stratifying the viruses by titration matrix or host origin (Table S2B, Fig. S4). That said, compared to the entire data set, limiting the analysis to H7N9 viruses did yield higher positive correlation coefficients for both Tr (*r* = 0.88 [0.52, 0.97], RS-*p* = 0.002) and Lg (*r* = 0.94 [0.73, 0.99], RS-*p* = 1e-4) titers (Fig. 1I; Table S2B). Collectively, we found that *in vitro* assessments of replication ability in Calu-3 cells cultured at 37°C are capable of informing data collected from both NW specimens and discrete tissues, but as expected, individual *in vitro* viral titer time points only accounted for some of the variability in the discrete viral titer time points measured *in vivo*.

Because the temperature of the mammalian nasal airways is lower than the core body temperature (16), standard replication kinetics evaluations were conducted in Calu-3 cells with a subset of viruses (*n* = 36) at both 33°C and 37°C (Table 1). Pearson correlations between peak NW titer and peak Calu-3 titer following culturing at 33°C were generally weaker compared to 37°C when employing the entire data set or when stratified by titration matrix or host origin (Table S2A and C; Fig. S4). However, in contrast to peak Calu-3 titer measurements, stronger correlations were identified between day 1 p.i. NW titer and mean viral titer at 24 h p.i. in Calu-3 cells cultured at 33°C than at 37°C in either the full data set (*r* = 0.54 [0.26, 0.74], RS-*p* = 6e-4, Fig. 1J) or following data stratification (Table S2C). Restricting these analyses to H7N9 or H1 variant subtype viruses further increased correlation coefficients (Fig. 1K to L; Table S2C). Compared with day 1 p.i. NW analyses, Pearson correlation coefficients employing mean viral titer at 24 h p.i. in Calu-3 cells cultured at 33°C were weaker when employing ferret titer data collected day 3 p.i. (either NW or tissue titers, Fig. S4), supporting the utility of 33°C titer measurements in predicting data collected early during *in vivo* infection (e.g., within 24 h p.i.).

### Mathematical modeling of *in vitro* infection in Calu-3 cells.

**FIG 2:**
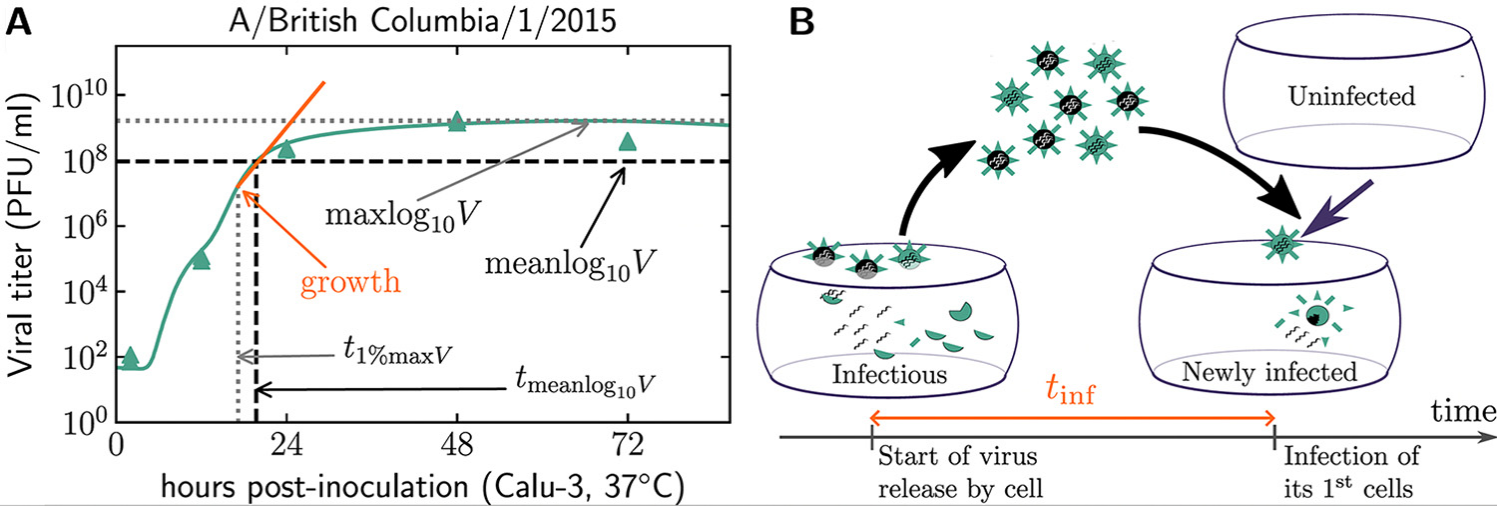
Representation of the MM-derived features of an *in vivo* viral time course. (A) An illustration of the key MM-estimated features shown against an example MM-predicted time course for infection of Calu-3 cells with A/British Columbia/1/2015 at 37°C. (B) A representation of the MM-estimated infecting time (*t*_inf_). All MM-derived quantities are described in more details in Table 2 and the Supplemental Methods.

The use of MMs is a useful and effective way to validate results from analyses based on experimentally measured titer alone, such as that presented above. It also offers enhanced analysis, between and beyond the sometimes sparsely collected time points. Data from Calu-3 infections were subjected to MM analysis to estimate key replication parameters and features of the viral titer time course for each virus (illustrated in Fig. 2 and described in Table 2). Since many parameters were not tightly constrained by the data, analyses are generally limited to looking at features of the MM-predicted time course, which were well informed by the data. A description of the MM and its associated analysis and limitations is provided in the Supplemental Methods.

**TABLE 2:** MM-derived key replication parameters and features of an *in vitro* viral time course

Parameter (units)^*a*^	Definition	Biological meaning^*b*^
maxlog_10_*V* (log_10_ viral titer)	Peak log_10_ viral titer	Peak titer present anytime 0 to 86 h; Calu-3 experimental time course calculates peak from 24 h, 48 h, or 72 h sampling times only
meanlog_10_*V* (log_10_ viral titer)	Mean log_10_ viral titer	Similar to an area under the curve measurement generated from experimental time course and then divided by the duration of the infection, but with increased accuracy since the entire MM-predicted time course (nearly continuous time points from 0 to 86 h) is used. It is largest for infections that reach peak titer more rapidly and/or sustain high titer over a longer duration and/or have a higher peak.
*t*_inf_ (h)	Time between start of virus progeny release by newly infected cell and infection of its first cell	No close equivalent can be estimated from experimental titer measurements alone. Similar to the serial interval in epidemiology. It is shortest for viruses that cause infected cells to produce and release progeny at a higher rate and/or whose progeny are more infectious (fewer virions required to cause a cell infection).
*t*_1%max_*_V_* (h)	Time to reach 1% of peak viral titer	No good equivalent in experimental time course because experimental measurement time points are typically too sparsely distributed in time to provide an accurate estimate of timings for viral titer milestones. This is shortest for the most rapidly progressing infections, but it can also be shortest for infections with a low viral titer peak that can be more rapidly attained.
*t*_meanlog10_*_V_* (h)	Time to reach peak mean log_10_ viral titer	Largely similar to *t*_1%max_*_V_* but since it is a different milestone, it can occasionally capture a slightly different timing.
growth (h^−1^)	Calu-3 titer growth rate	This is similar to calculating the slope between pairs of exptl log_10_ viral titer time points but provides a more accurate/reliable estimate since the MM analysis uses all exptl viral titer measurements to inform this measure. A virus with a higher growth rate claims more cells per infected cell per hour; this measure is equivalent to the basic reproductive number divided by the life span of an infected cell.

a All parameters pertain to the MM-simulated time course (0 to 86 h p.i.) derived from Calu-3 *in vitro* generated data and were extracted from the MM-predicted most likely viral titer time course given the experimental data, rather from the data itself.

b Added benefit of MM parameter relative to viral titer-only time course, both of which are inevitably measured at sparse experimental time points.

We first examined how well *in vitro*-generated Calu-3 data aligned with analogs from the MM-predicted values (e.g., from time points measured 24, 48, and 72 h p.i.). As expected, strong correlations were observed between the MM-predicted and experimentally measured peak titers (*r* = 0.99, [0.97, 0.99], RS-*p* = 1e-16) and 24 h p.i. titer (*r* = 0.95, [0.91, 0.97], RS-*p* = 1e-16) for infections in Calu-3 cultured at 37°C (Fig. S5A to F). Comparable correlations between MM-predicted and experimentally measured titers were observed at 33°C, and when data were stratified by titration matrix (Table S3A). Substitution of these MM-predicted values into analyses with ferret titer measures (Table S3A; Fig. S5G to I) yielded correlations generally comparable to those found with the Calu-3 experimental measurements (reported in Fig. 1 and Table S2A and B).

We next explored the utility of other MM-predicted values not obtainable by experimental *in vitro* study alone to offer enhanced predictive ability of *in vivo*-generated ferret measures. Unlike peak titer assessments, which are determined by sampling culture supernatant at specific times p.i. and therefore may miss actual titer peaks, MM can predict the maximum titer over the entirety of the time course (in this instance, 0 to 86 h). When employing the entire data set, both peak ferret NW titers (*r* = 0.32 [0.05, 0.54], RS-*p* = 0.02) and day 3 p.i. nasal turbinates (NT) titers (*r* = 0.52, [0.27, 0.70], RS-*P* = 2e-4) were positively correlated with the MM-predicted peak titer (maxlog_10_*V*, Table S3B) when cultured at 37°C, in alignment with similar correlations observed when employing peak Calu-3 titer. With few exceptions, MM-predicted and experimentally measured Calu-3 peak titer offered similar predictive benefits against all ferret parameters examined (Table S2A and B versus 3B), suggesting that collected time points during *in vitro* experimentation generally encompass the maximum titer possible from the culture. Comparable correlations were also observed when employing the MM-predicted mean viral titer over 0 to 86 h (meanlog_10_*V*), rather than peak titer (Table S3B).

Finally, we examined the choice of discrete time points within the replication curve. While 24 h represents the earliest time point examined in the Calu-3 infections in this study above baseline measurements, selected studies will report viral titers from the initial rounds of viral replication, such as 12 h or 16 h p.i.; MM-predicted titers at these times exhibited equivalent or weaker correlations from those at 24 h (Table S3C), at either temperature examined. Collectively, these analyses highlight the ability of the MM to inform time-point selection criteria and support the utility of peak titer and 24-h time point measurements generated *in vitro* to capture key aspects of viral load over the course of an infection initiated with an MOI of 0.01 infectious units/cell.

### Correlations between mathematical metrics *in vitro* and raw titers *in vivo*.

**FIG 3:**
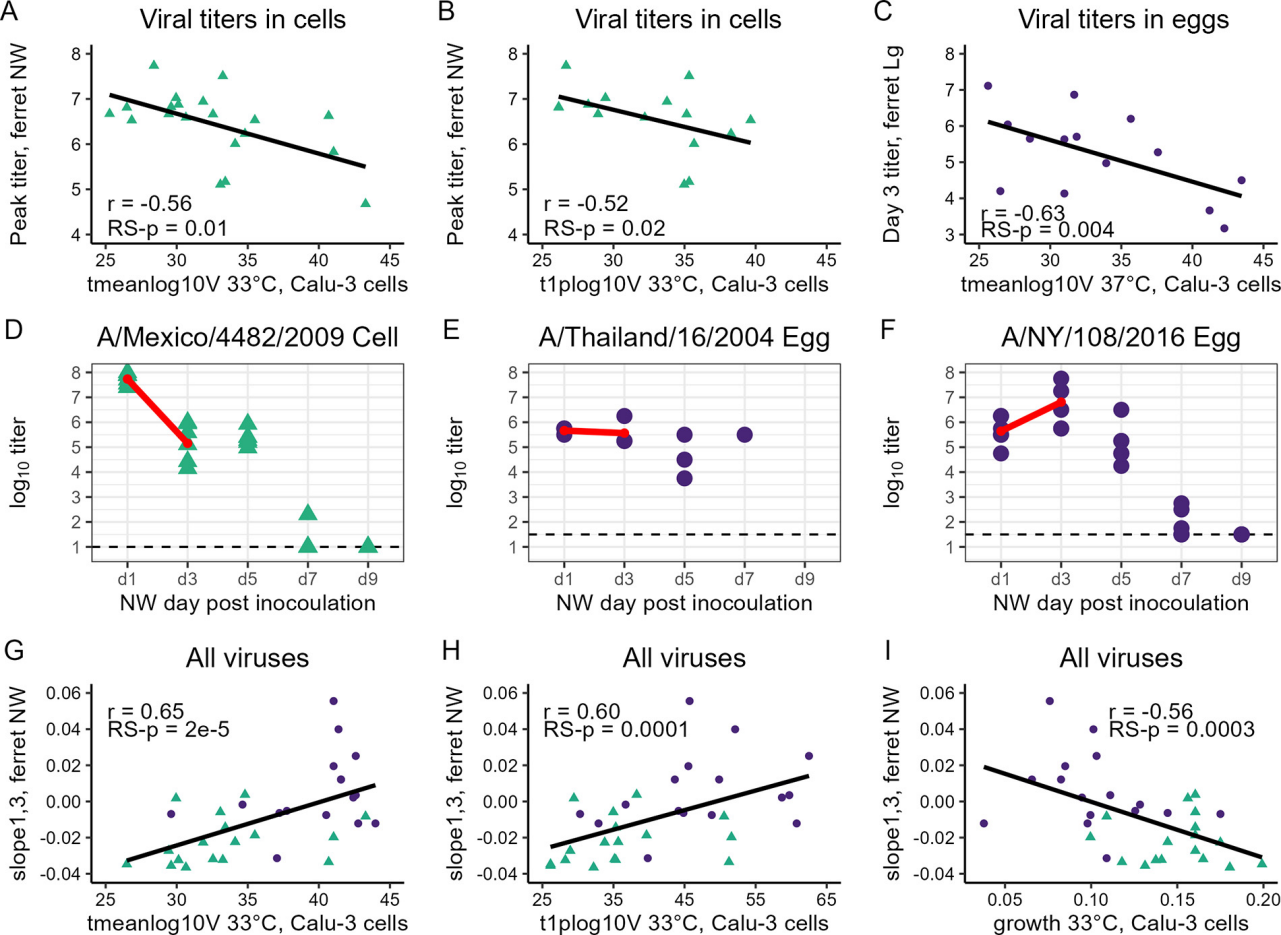
Correlations between ferret nasal wash and MM-predicted parameters. Best-fit line determined by least-squares regression; *r* values are from Pearson correlation tests (see Supplemental Table 3). Viral titers correspond to the log_10_ of the viral titers, measured in EID_50_/mL (NW) or EID_50_/g (Lg) (viral titration in eggs, circles) or PFU/mL (NW) (viral titration in cells, triangles); sample titration matrix in mixed data sets is specified in Table 1. (A to C) Peak ferret NW versus *t*_meanlog10_*_V_* (33°C) (A) or t_1%max_*_V_* (33°C) (B) for viral titers determined in cells only and day 3 p.i. lung versus *t*_meanlog10_*_V_* (37°C) (C) for viral titers determined in eggs only. (D to F) Representative graphs of ferret NW specimens; red line presents slope_1,3_ generated for representative viruses showing decay (D), no change (E), or growth (F) between mean log_10_ NW titers day 1 and 3 p.i. Horizontal line depicts titration limit of detection for each matrix. (G to I) Pearson correlation tests of slope_1,3_ versus MM-predicted parameters t_meanlog10_*_V_* (G), *t*_1%max_*_V_* (H), and growth (I) at 33°C.

After confirming that MM parameters that emulate data points collected during experimental infection offered similar predictive benefit to *in vivo* titer measurements as data generated experimentally, we next examined the predictive ability of parameters associated with the timing of viral growth and spread *in vitro*, which cannot be accurately determined without a MM. The MM can identify the time to reach intermediate (pre-peak) viral titer milestones, including the MM-predicted time for the viral titer to reach 1% of peak titer (labeled *t*_1%max_*_V_*) or time to reach the log_10_ mean titer over 0 to 86 h (*t*_meanlog10_*_V_*). For the viral titers determined in cells, negative correlations were observed between ferret NW (peak or day 1 p.i. titers) and either *t*_1%max_*_V_* or *t*_meanlog10_*_V_*, which were consistently stronger for infections cultured at 33°C than 37°C (Fig. 3A and B; Table S3D). This suggests that viruses that grow more rapidly *in vitro* (i.e., achieve these infection progression milestones more quickly) are detected at higher titer in NW specimens *in vivo*. These same correlations were weaker for the viral titer determined in eggs and not consistently detected for tissues collected at day 3 p.i., at either culture temperature, with few exceptions. The most notable exception was the strong negative correlation, among viruses titrated in eggs, between ferret lung (Lg) titer at day 3 p.i. and either the time to reach 1% peak titer, time to reach the mean log_10_ titer, or the infecting time (i.e., the time for an infectious cell to cause the infection of one another, similar to the serial interval in epidemiology, labeled *t*_inf_) in MM-predicted infections at 37°C (Fig. 3C; Table S3D). This suggests that viruses titrated in eggs that grew more rapidly (e.g., shorter time to reach pre-peak milestones) in Calu-3 were strongly associated with higher ferret Lg titers at day 3 p.i., but not (or less so) with higher peak or day 1 p.i. NW titers.

### Correlations between mathematical metrics *in vitro* and titer growth/decay *in vivo*.

We next explored whether measures of infection progression in ferrets, rather than direct titer measurements used thus far, would provide novel or more robust comparison to the MM-predicted metrics. Unfortunately, due to various limitations inherent to both the shape of the viral titer time course and the sparsity of data collected over the course of infection in ferrets (discussed in Supplemental Methods), we were not able to apply the MM analysis performed in Calu-3 cells to the ferret viral titer time courses. As an intermediate analysis, between raw titer and full MM-estimated infection time courses, we estimated the rate of decay (negative slope) or growth (positive slope) of the NW titer in ferrets for every sampling interval between days 1 to 7 p.i., as described in the Supplemental Methods (shown therein and in Fig. 3D to F). The MM-predicted measures of infection progression *in vitro* were highly correlated with the rate of growth or decay of the log_10_ NW titer between day 1 and 3 p.i. (labeled slope_1,3_ to indicate the slope between these sampling points). Specifically, viruses with a shorter time to reach pre-peak milestones (*t*_1%max_*_V_*, *t*_meanlog10_*_V_*, *t*_inf_) and a more rapid initial growth rate (labeled growth) in Calu-3 cells were all associated with the greatest decay in ferret NW titer between day 1 and 3 p.i., for viral titer determined in both eggs and cells, at either temperature (Fig. 3G to I). In other words, viruses whose infections progressed most rapidly in Calu-3 cells also progressed most rapidly within ferrets, so much so that viral titer had already peaked and was on its way down soon after day 1 p.i. in ferrets. Significant correlations were maintained among viruses of avian, but not mammalian, origin (Table S3E). Of note, among the mammalian viruses in this study, 21/24 had negative slope_1,3_ values, indicating NW titer decay between day 1 and 3 p.i., while viruses of avian origin were equally split between positive and negative slope_1,3_ values (*n* = 14 each), indicating greater uniformity in this parameter among mammalian viruses (RS-*p* = 0.007 by Fisher’s exact test). In contrast, instantaneous growth/decay rates between other pairs of time points within these data set (e.g., between day 3 and 5 p.i., slope_3,5_) provided poorer correlations than slope_1,3_ (data not shown). Taken together, these findings support the utility of employing infection progression parameters to link virus replication *in vitro* and *in vivo* but highlight the challenges in data interpretation when employing heterogenous viruses possessing differing levels of mammalian adaptation.

### Exploring virus behavior under different temperature conditions.

**FIG 4:**
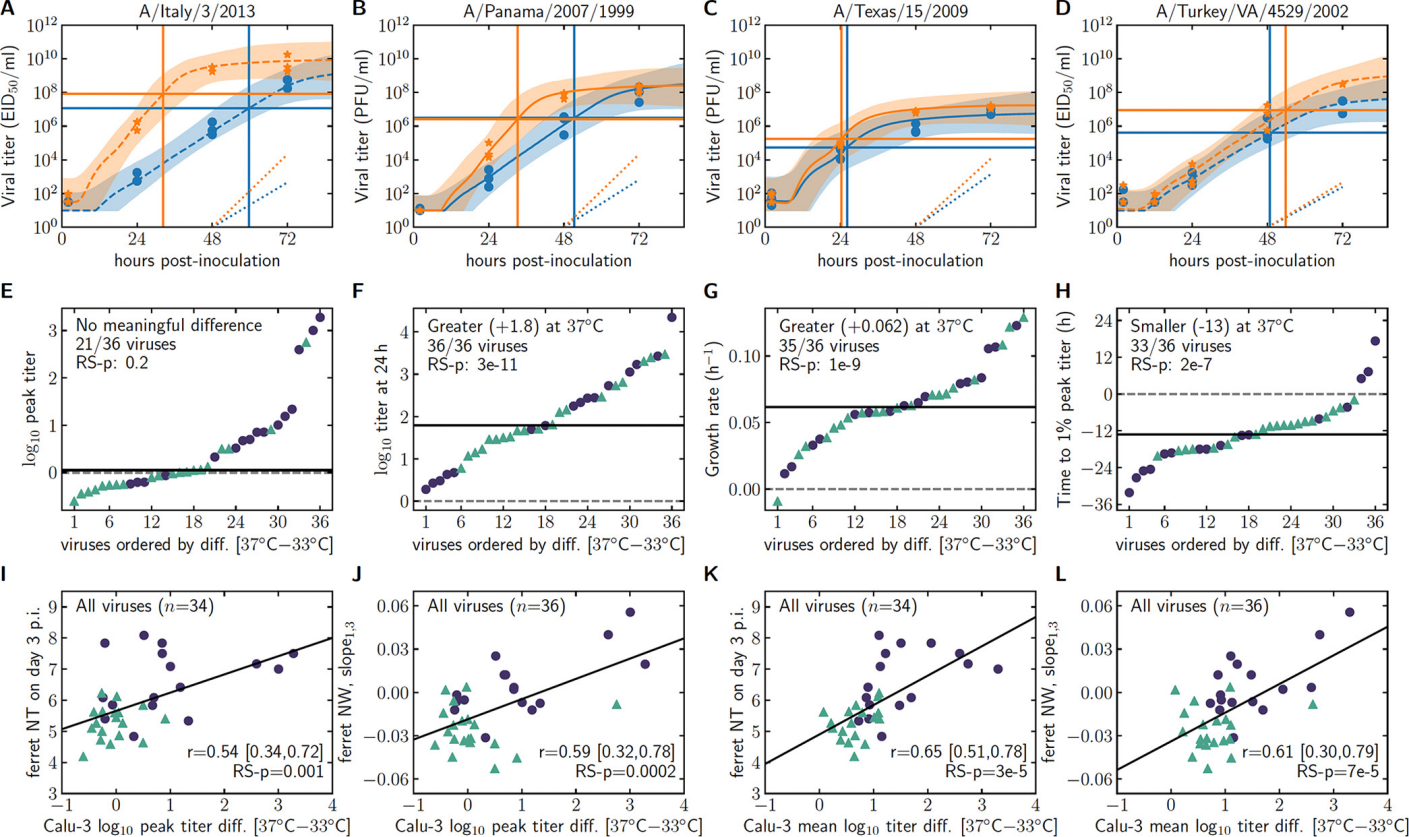
Comparison of the effect of temperature sensitivity on MM-predicted parameters. (A to D) Time course of experimental and MM-simulated *in vitro* IAV infections in Calu-3 cells. Cells were infected in triplicate with the viruses shown at either 37°C (orange, stars) or 33°C (blue, circles). Experimental measurements are shown as symbols. The MM-predicted time course is shown as a solid (titer determined in cells) or dashed (titer determined in eggs) line with 95-percentile bounds at each time point (shaded regions). Horizontal and vertical solid lines represent 1% of the MM-predicted peak viral titer and the time at which it occurs (*t*_1%max_*_V_*), respectively. The dashed diagonal lines in the bottom right of each graph are a visual representation of the growth rate of the virus (growth, see Table 2). Time courses for all viruses are shown in the Supplemental Methods. (E to H) Difference in MM-derived quantity at 37°C minus that at 33°C, computed for all viral titers determined (*n* = 36) in eggs (circles) or cells (triangles), ordered from smallest (or most negative) to largest (or most positive). The horizontal dashed line corresponds to zero (no difference), and the horizontal solid line corresponds to the median difference over the 36 viruses, also indicated in parenthesis [e.g., “Greater (+1.8)]”. (I to L) Correlations are between the indicated ferret titer measurement (*y* axis) against the difference in MM-derived quantity at 37°C minus that at 33°C (*x* axis). Best-fit line determined by least-squares regression; *r* values are from Pearson correlation tests.

Slower infection progression or lower viral titer yield at 33°C than at 37°C could be indicative of poor adaptation of a virus to the cooler temperature of the upper mammalian airways. To investigate this possibility, we used the MM-predicted infection time course and parameters of 36 viruses for which replication kinetics were assessed in Calu-3 cells at both 37°C and 33°C (Fig. 4A to D; Supplemental Methods). The difference in each MM-predicted quantity at 37°C minus that at 33°C was computed across the 36 viruses and then ordered (1st to 36th) from smallest (most negative, indicating that the quantity is lower) to greatest (most positive, indicating that the quantity is larger) at 37°C compared to that at 33°C (Fig. 4E to H; Supplemental Methods).

The MM-predicted titer at 24 h and 36 h p.i., during the intermediate, pre-peak growth phase of infection in Calu-3, and the mean log_10_ titer were larger, and the infecting time (*t*_inf_) was shorter for all 36 viruses at 37°C compared to 33°C. Similarly, the MM-predicted titer at 12 h and 16 h p.i. and the Calu-3 titer growth rate were higher, and the time to reach mean log_10_ titer or 1% peak titer was shorter at 37°C compared to 33°C, in all but at most 3 out of 36 viruses (Fig. S3). In many cases, the differences were quite striking and are most likely biologically significant. For example, for 18 out of the 36 viruses, the MM-predicted titers at 24 h p.i. were at least 63 times higher (10^+1.8^ times greater) and titer grew at least 15% more rapidly (10^+0.062^ times greater), such that the 1% of peak titer was reached at least 13 h earlier (*t*_1%max_*_V_*) at 37°C compared to 33°C (Fig. 4F–H; Supplemental Methods). In contrast, the MM-predicted and experimentally measured peak Calu-3 titer and titer at 72 h p.i., the typical time of titer peak, were lower for 10 versus higher for 26 out of 36 viruses, at 37°C compared to 33°C, with a likely experimentally unmeasurable median difference of 1.5-fold (a log_10_ titer difference of 0.1), with the interesting exception of 4 viruses (e.g., A/Turkey/VA/4529/2002, Fig. 4D) for which peak titer at 37°C was more than 100-fold higher than at 33°C, whereas the exponential titer growth rate was comparable at either temperature. Taken together, these observations suggest rather strongly that while peak titer is generally unaffected by the culture temperature, higher temperature generally hastens infection progression.

As the temperature of the ferret respiratory tract is inclusive of both 33°C and 37°C, we next investigated if differences in temperature sensitivity *in vitro* could serve as predictive measures of *in vivo* replicative fitness (Table S3F; Fig. 4I to L). Interestingly, the difference in MM-predicted peak titer in Calu-3 (maxlog_10_*V*) at 37°C minus that at 33°C, a quantity that was not consistently and generally only weakly affected by temperature (Fig. 4E), correlated most strongly with slope_1,3_ (*r* = 0.59 [0.28, 0.80], RS-*p* = 0.2e-4), ferret NT titer at day 3 p.i. (*r* = 0.54 [0.35, 0.76], RS-*p* = 0.001), and ferret NW titer at day 1 p.i. (*r* = −0.43 [−0.66, −0.10], RS-*p* = 0.003) (Fig. 4I to J; Table S3F). There were also strong correlations between the difference in the MM-predicted mean log_10_ viral titer in Calu-3 (meanlog_10_*V*) at 37°C minus that at 33°C, a quantity that was significantly affected by temperature (Supplemental Methods), and both the NT titer at day 3 p.i. (*r* = 0.65 [0.5, 0.77], RS-*p* = 3e-5) and slope_1,3_ (*r* = 0.61 [0.22,0.78], RS-*p* = 7e-5) (Fig. 4K to L). Together, these correlations suggest that viruses that experience the largest decrease in their peak titer or total titer yield (mean log_10_ titer) *in vitro* in Calu-3 cells as a result of the decreased temperature from 37°C down to 33°C grow the slowest in ferrets, resulting in lower NW titer at day 1 p.i., with continued growth to day 3 p.i. (more positive slope_1,3_), leading to higher, detectable titer in the NT by day 3 p.i. Collectively, these findings support the use of a MM toward understanding the behavior of IAV strains *in vitro*, which can subsequently inform *in vivo* phenotypes of each virus, and underscore the utility of assessing IAV replicative ability at multiple physiologically relevant temperatures.

### Correlation with nonviral titer contributing factors.

The capacity for high-titer virus replication in mammals represents just one of many features that contribute to zoonotic viruses overcoming host range restrictions; these can include molecular determinants of virulence with known roles in modulating virus replication, receptor binding, pH fusion, and other critical properties (17). While the scope and number of viruses in this study prohibited an exhaustive examination of all features likely to contribute, and analyses were limited to data stratified by titration matrix, we employed representative examples of key features often evaluated during *in vivo* risk assessments in the context of *in vitro* study.

**FIG 5:**
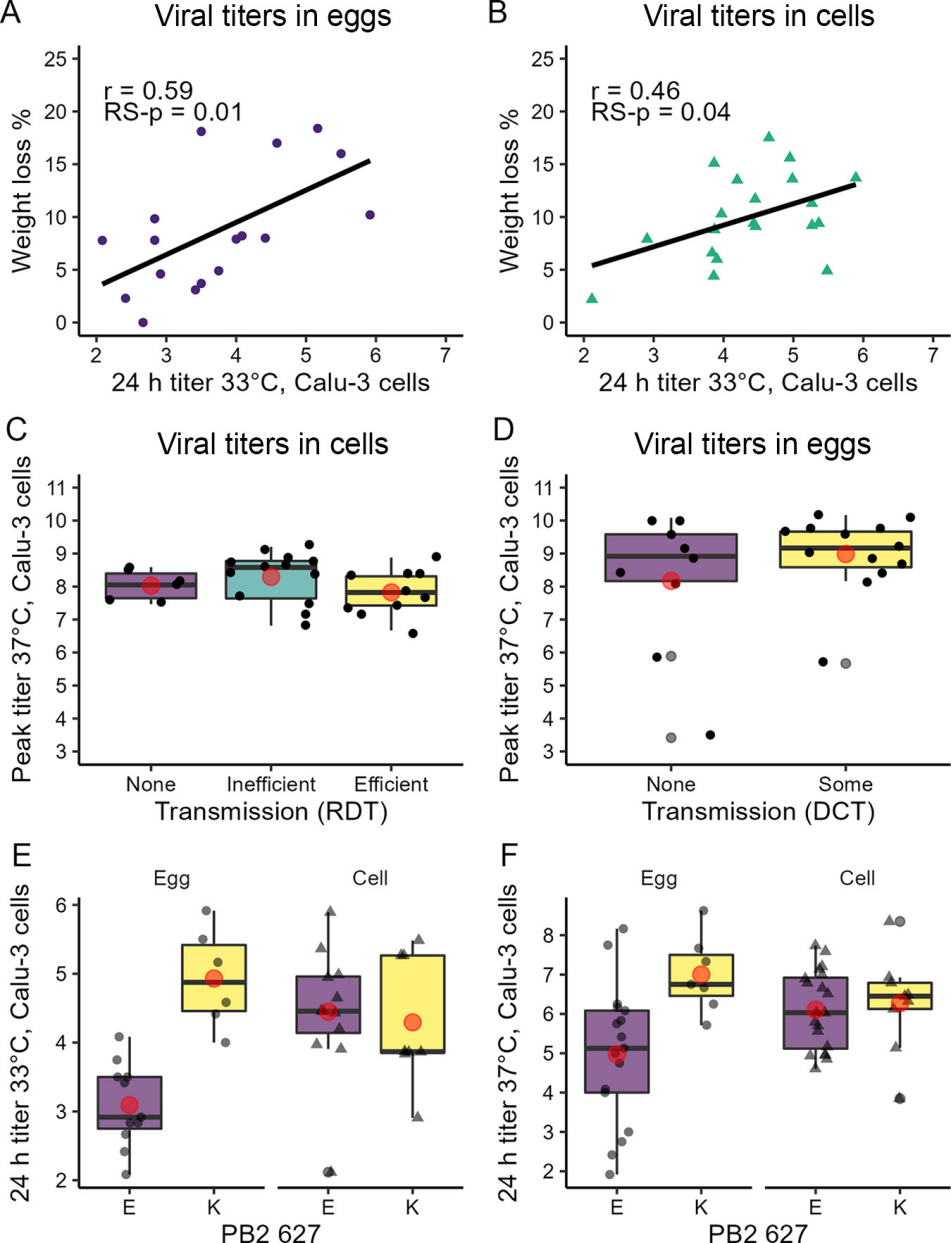
Correlations between ferret nasal wash, PB2 627, weight loss, and 33°C Calu-3 viral titers following IAV infection. Best-fit line determined by least-squares regression; *r* values are from Pearson correlation tests (see Table S2). Viral titers correspond to the log_10_ of the viral titers, measured in EID_50_/mL (viral titration in eggs, circles) or PFU/mL (viral titration in cells, triangles); sample titration matrix is specified in Table 1. (A and B) Percent mean maximum ferret weight loss versus 24-h Calu-3 titer (33°C) for viral titers determined in eggs (A) or cells (B). (C) Box and whiskers plot of peak Calu-3 titer (37°C) among viruses exhibiting transmission in 0/3 pairs in a respiratory droplet transmission (RDT) ferret model (none), transmission in 1/3 or 2/3 pairs (inefficient), or transmission in 3/3 pairs (efficient) among viral titers determined in cells. (D) Box and whiskers plot of peak Calu-3 titer (37°C) among viruses exhibiting transmission in 0/3 pairs in a direct contact transmission (DCT) ferret model (none), or transmission in at least one ferret pair (some). (E and F) Box plot of 24-h Calu-3 titer at 33°C (E) and 37°C (F) for viruses bearing an E or K at PB2 627; data points represent individual viral titers determined in eggs or cells as specified in Table 1. Red dot in C to F plots signifies mean.

Measures of virus pathogenicity in ferrets (such as weight loss and lethality) are considered in risk assessment rubrics, but it was unclear if Calu-3 IAV replication data could inform or otherwise contribute to our understanding of these properties. Peak mean weight loss in ferrets was more highly correlated with *in vitro* titer measurements collected at 24 h rather than with peak Calu-3 titer (Table S2D), with stronger correlations observed following culturing at 33°C compared with 37°C. At the lower temperature, the 24-h Calu-3 titer for the viral titer determined in eggs (*r* = 0.59 [0.16, 0.84], RS-*P* = 0.01) and cells (*r* = 0.46 [0.02, 0.75], RS-*p* = 0.04) was both correlated with peak mean weight loss in ferrets (Fig. 5A and B). Assessments correlating the magnitude of IAV titers *in vitro* to a lethal phenotype *in vivo* were limited, because only six strains exhibited >50% lethality (all viruses titrated in eggs). However, compared with 18 nonlethal viruses titrated in eggs, viruses exhibiting >50% lethality in ferrets replicated to marginally higher peak titer in Calu-3 cells (median [95% CI] 10^9.4^ [10^8.1^, 10^10^] EID_50_/mL versus 10^8.7^ [10^4.6^, 10^10^] EID_50_/mL) and higher titer at 24 h p.i. (median [95% CI] 10^7.3^ [10^5.0^, 10^8.7^] EID_50_/mL versus 10^5.3^ [10^2.2^, 10^7.9^], fold difference 10^2.1^ [10^1.5^, 10^5.8^] EID_50_/mL) at 37°C.

Virus transmissibility cannot be modeled outside a living host, and both transmissible and nontransmissible IAV replicate robustly in Calu-3 cells (18). Prior studies have identified links between donor viral load and transmissibility (19) but have not examined the utility of *in vitro* measures in this context. Correlations were not detected between virus transmissibility (in either a respiratory droplet or direct contact transmission model) and absolute Calu-3 viral titer (peak titer or 24-h titer cultured at 37°C, or 24-h titer cultured at 33°C) (Fig. 5C and D).

Residue 627 in PB2 has long been recognized as a critical determinant of host range for IAVs (20), with known roles in modulating mammalian pathogenicity and transmissibility, polymerase activity, and temperature sensitivity (21). For infections of Calu-3 cells, the viral titer determined in eggs with a lysine (K) at this position reached higher titer by 24 h p.i. than viruses bearing a glutamic acid (E) at both culture temperatures: a median [95% CI] 24-h titer of 10^5.2^ [10^4.1^, 10^5.9^] EID_50_/mL versus 10^2.9^ [10^2.2^, 10^4.0^] EID_50_/mL at 33°C (fold difference 10^1.9^ [10^0.33^, 10^3.5^] EID_50_/mL) and 10^6.8^ [10^5.3^, 10^8.7^] EID_50_/mL versus 10^5.0^ [10^2.1^, 10^8.0^] EID_50_/mL at 37°C (fold difference 10^2.1^ [10^−1.6^, 10^5.8^] EID_50_/mL) (Fig. 5E and F). This was not the case for viruses titrated in cells where no meaningful difference was observed. While viruses bearing E627K did reach higher peak titers *in vitro*, the presence of the E627K mutation alone was not associated with significantly higher titers in ferret NW (peak or day 1 p.i.) or day 3 p.i. in NT (Table S2D).

## DISCUSSION

Pandemic preparedness efforts encompass a diverse array of approaches to understand not only the properties of novel influenza viruses but also the host and environmental contexts in which these viruses emerge (1, 2). As such, *in vitro* and *in vivo* assessments presented in this study represent just one of a multitude of concurrent activities performed when assessing the pandemic potential of influenza viruses. As laboratory assessments of novel and emerging influenza viruses are conducted worldwide, it is critical to understand how different experimental protocols can inform and improve our ability to contextualize results, to achieve the highest confidence possible in conclusions drawn from concurrent *in vitro* and *in vivo* evaluations. Here, we use viral titer measurements from IAV infections *in vitro* to assess the extent to which they correlate with *in vivo* viral titer measurements in the respiratory tract of ferrets following infection and employ mathematical modeling to examine in finer detail the replication parameters not apparent by traditional virology analyses alone.

The data presented in this study represents an analysis of *in vitro* and *in vivo* data generated under consistent laboratory experimental protocols representative of those employed in risk assessment activities, an approach that helps reduce the effect of confounders when comparing data between different laboratory groups (7, 22). Due to extensive heterogeneity in both *in vitro* (3) and *in vivo* (5) protocols associated with influenza virus risk assessment activities, analyses investigating different cell types (such as A549 or NHBE cells), titration methods (such as TCID_50_), and ferret specimens (such as nose/throat swabs) were not included in this study. Calu-3 cells are highly permissive to IAV infection and support high-titer replication of a range of human and zoonotic IAVs without the addition of exogeneous trypsin, making them well suited to evaluate the heterogenous panel of viruses in this study (18). Both titer and infection progression MM-predicted parameters reported here are specific to Calu-3 cells grown under Transwell conditions, and would likely vary should the culture method or cell type be changed. Despite these limitations on the *in vitro* culture condition and the MM analysis, the utility of MM to validate time point selection during experimentation and to identify features of the replication curve (with regard to both viral titer and infection progression kinetics) that offer the highest predictive value to *in vivo*-derived measurements highlights the benefit that this analysis approach can contribute to the interpretation of data generated for risk assessment purposes.

Our data set included 52 contemporary IAV evaluated in both *in vivo* and *in vitro* models via consistent experimental protocols. An important limitation within this data set was the use of two different titration matrices (eggs and cells). There were numerous instances where high-ranking correlations were identified between *in vitro* and *in vivo* measures for viruses titrated in one titration matrix that were low ranking in the other matrix (notably with regard to measures of infection progression). We suspect this is because zoonotic viruses of avian origin tend to be titrated in eggs while mammalian-origin viruses tend to be titrated by plaque assay, as eggs are more sensitive in detecting the presence of avian-origin viruses, which may not grow well in mammalian cells. In contrast, many human and swine influenza viruses are preferentially titrated in mammalian cells (typically MDCK cells), due to robust growth in this matrix and to avoid the potential for egg-adapted mutations (23). In support of this, stratifying the data set by virus origin rather than titration matrix when possible resulted in generally comparable correlation coefficients and maintenance of statistically significant findings, although this was not possible in all analyses. As such, we cannot fully separate the contributing role of titration matrix versus host origin when reporting observed correlations. Nonetheless, considering the dual use of both egg and cell titration of IAV in the field, approaches such as this to better control for and interpret data generated from multiple titration matrices represent a critical and overdue effort.

The virologic and phenotypic heterogeneity among viruses in these data sets represented an additional challenge for comparative study. Striking differences in exponential growth or decay of replicating virus in ferret NW specimens depending on virus host origin (as supported by slope_1,3_ measurements) further support the idea that mixed data sets inclusive of both mammalian- and zoonotic-origin IAV may exhibit host-specific features during replication *in vivo*. Of note, even the contribution of specific molecular determinants of virulence can be challenging to assess in data sets with viruses containing substantial genetic diversity. For example, when cultured at 33°C, the PB2 627K mutation was associated with a higher growth rate in Calu-3 among viruses titrated in eggs (0.13 [0.11, 0.17] h^−1^ 627K versus 0.095 [0.045, 0.11] h^−1^ 627E) but not among viruses titrated in cells (0.16 [0.12, 0.2] h^−1^ 627K versus 0.15 [0.08, 0.19] h^−1^ 627E); other MM-predicted parameters (e.g., *t*_1%max_*_V_*) exhibited similar, though less striking, trends. In the case of PB2 E627K, we examined one mutation in isolation (Table S2D); it is known that many amino acid substitutions can functionally compensate for one another (24) and that polygenic traits such as pathogenicity and transmissibility are often a reflection of specific mutations present at multiple amino acid residues (25). Limiting analyses to genetically related viruses (e.g., H7N9) often yielded higher correlation coefficients, although this could be at least in part due to the smaller number of viruses in these analyses; nonetheless, this study provides a framework for further investigation of predictive roles for specific molecular features *in vitro* in predicting *in vivo* behavior should viruses that possess reduced genetic diversity be employed.

As similar analyses have not previously been conducted, we designed this work to be exploratory in nature, intended to identify avenues and analysis strategies for further investigation rather than to provide definitive findings. Considering the substantial number of metrics evaluated throughout this study (from both raw Calu-3 data and MM derived, many of which were ultimately not included in this report), we do not report the absolute statistical significance of any correlation to avoid overinflation of reported significance. Rather, we sought to focus on biologically meaningful relationships present between parameters of the viral replication curve *in vitro* and multiple sample types *in vivo*. Linear associations (identified via Pearson correlations) between viral titers obtained *in vitro* and *in vivo* were frequently present, though correlations rarely exceeded *r* = 0.5 (Table S2), even among our comparisons with the highest statistical significance. Of note, analyses repeated using rank correlations were found to have relative significance (RS-*p*) and strength (*r*) largely similar to those obtained with the linear correlations (see Supplemental Methods). Calculation of predictive power scores (PPS) was employed as a complementary analysis approach to Pearson correlations, as this asymmetric nonlinear index can explore predictive nonlinear and asymmetric relationships between *in vitro* and *in vivo* data not possible by linear correlation analyses alone (26, 27). PPS analysis of viral titer data *in vitro* generally agreed with the strength of correlations obtained with Pearson correlations (Table S4). Collectively, these analyses demonstrated that viral titer outcomes *in vitro* are associated with viral titers from both nasal wash specimens and discrete tissues collected *in vivo* during the acute phase of infection, even when working with heterogeneous viruses, but can be dependent on the IAV under investigation, culture temperature *in vitro*, and sampling time and specimen type *in vivo*. Furthermore, these analyses identified novel areas of investigation to pursue metrics associated with infection progression in both *in vitro* and *in vivo* settings.

A MM, by its structure and the biological assumptions it encodes, constrains what range of viral titer measurements are possible over the course of an infection. Experimental measurements then provide further constraints on the shape of the MM-predicted curves, which the MM translates into constraints on its parameters (e.g., rate of virus production by each infected cell, life span of virus-producing cells, etc.). This allows a MM that is well informed by experimental measurements to successfully interpolate and predict unobserved quantities, smooth over experimental variability, and isolate different facets of the replicative fitness of a virus (13, 28). The *in vitro* data in the analyses considered herein were sparser (titers collected only at 1 to 2 h, 24 h, 48 h, and 72 h) than what is ideally required for MM analysis and often did not include data points during the viral decay phase following titer peak. As such, the MM-predicted viral titer curves were generally well constrained, but less so for the MM parameters.

Mathematical modeling provides an analytical and quantitative approach to examine the magnitude and impact of IAV replication efficiency relative to infection temperature in a more analytical and quantitative way than is possible with standard experimental measures. While experiments only allow us to compare the difference in titers at specific times, MM-derived quantities such as the infection growth rate or the time to reach 1% of peak titer suggested that higher temperatures generally increased the progression rate of the infection (how quickly peak titer is reached) more so than it increased the peak titer (Supplemental Methods). Interestingly, with regard to temperature sensitivity, viruses for which MM-derived peak and mean log_10_ Calu-3 titers decreased the most when the infection temperature was decreased from 37°C to 33°C were generally found at higher titers at day 3 p.i. In ferret NT, the tissue specimen exposed to the coolest temperatures (Fig. 4I and K) (16, 29). This same drop in peak and mean log_10_ Calu-3 titers at lower infection temperature was also associated with lower day 1 p.i. NW titers (Table S3F) and a larger (more positive) slope_1,3_ (Fig. 4J and L). Taken together, these findings suggest that viruses whose yield is most compromised at lower temperatures *in vitro* tend to grow more slowly *in vivo*, peaking at day 3 p.i., the day tissue titers were collected, rather than day 1 p.i. It is also of interest that this was more pronounced among viruses titrated in eggs (largely comprised of avian-origin strains) and not viruses titrated in cells (predominantly mammalian-origin strains), suggesting a potential difference in host adaptation captured by these analyses. To our knowledge, this is the first time a MM has been used to analyze infections conducted at different temperatures and identifies several potential areas of future inquiry. Unfortunately, because individual MM parameters were not well constrained by the analysis, we were not able to ascribe the effect of lower temperature to one or more of the virus replication steps captured by the MM parameters. Despite this, identification of heightened predictive power of 33°C Calu-3 data for upper respiratory tract specimens collected *in vivo* highlights the utility of employing culture temperatures emulative of the human upper respiratory tract when conducting risk assessment evaluations *in vitro*.

While it would have been highly desirable, we were not able to expand the same MM analysis to the *in vivo* infection data. This is in part because most of the ferret infection time course data considered herein either were already at peak titer at the first NW sample collected and decreased thereafter or had a single NW sample measurement collected before reaching peak titer (see Supplemental Methods). While the timing of this sampling is a reflection of appropriate anesthesia schedules for laboratory animals (30), these data lack key kinetic information, specifically the rate at which infection expanded in the host, i.e., the viral titer up-slope, which is required by the MM to robustly extract the replication efficacy of each strain. Furthermore, administration of a high-titer inoculum is necessary when evaluating pathogenicity of IAV with unknown 50% ferret infectious doses, but collection of the first NW specimen 24 h p.i. means that the presence of residual inoculum in this sample cannot be fully ruled out. Despite these challenges, we calculated the growth or decay rate of the ferret NW titer between pairs of measurement time points (see Materials and Methods) and identified one (slope_1,3_), which showed strong correlations with mathematical quantities characterizing infection kinetics *in vitro*. Our findings indicate that viruses exhibiting the most rapid growth *in vitro* were associated with the greatest decay between NW specimens collected day 1 and 3 p.i., likely driven by mammalian-origin viruses exhibiting robust replication in a mammalian cell line and productively infecting the ferret upper respiratory tract early after infection due to strong binding to α2,6 linked sialic acid receptors. Poorer correlation with measures of titer growth or decay in ferret NW beyond day 3 p.i. may be attributed to elicitation of diverse innate immune responses and other confounders (31); it should be noted that NW specimens are inclusive of multiple independent sites of virus replication throughout the ferret upper respiratory tract, where infection likely proceeds at different rates, peaking at different times (32). MMs incorporating experimental data from *in vitro* experimentation have been used to inform refinement of *in vivo* models and kinetics of host response elicited following viral infection, most frequently in mouse models (33, 34), highlighting the need to perform similar analyses in the ferret. As public health efforts increasingly rely on data generated from the ferret model to prevent and mitigate influenza virus infection in humans, it is prudent to similarly explore the use of MMs for data generated in this species, especially as *in vitro* assessments are often employed before *in vivo* experimentation in adherence to the 3Rs (replacement, reduction, and refinement) of animal research when employing small mammalian models (35).

This study emphasizes the need for *in vivo* assessments so long as *in vitro* experimentation cannot fully recapitulate or predict *in vivo* virulence phenotypes. That said, it is possible that, under yet-to-be-identified protocols for both *in vitro* and *in vivo* experimentation, additional correlates between these experiments could be identified and the predictive power of *in vitro* data could be significantly increased. For example, the adoption of a more accurate measure of virus sample infectivity (36) or identifying and then better managing *in vitro* factors that undermine reproducibility across *in vitro* infection experiments (37) could improve correlations between within-host and *in vitro* infections (38). Ultimately, this study identifies previously unrecognized correlates between data generated *in vitro* and *in vivo* for the purpose of influenza A virus risk assessment and highlights potential future areas of investigation to more quantifiably apply *in vitro* data toward pandemic preparedness efforts.

## MATERIALS AND METHODS

### Viruses.

Influenza A viruses evaluated *in vivo* and *in vitro* and included in this study are listed in Table 1. Virus stocks were either propagated in the allantoic cavity of 10- to 11-day-old embryonated chicken eggs, or MDCK cells, as described in the provided references. Infectivity titers following *in vivo* or *in vitro* inoculation were generated by determination of the EID_50_ or PFU titer in MDCK cells (London line) by standard plaque assay, as indicated in Table 1. As specified in the references, zoonotic influenza viruses were manipulated under biosafety level 3 containment, including enhancements as required by the U.S. Department of Agriculture and the National Select Agent Program (39).

### Ferret data.

All *in vivo* data presented here were performed under the guidance of the Centers for Disease Control and Prevention’s Institutional Animal Care and Use Committee and were conducted in an Association for Assessment and Accreditation of Laboratory Animal Care International-accredited animal facility. Ferret pathotyping data were published previously as indicated by references in Table 1. Briefly, ferrets (5 to 12 months of age, Triple F Farms, Sayre, PA), serologically negative to circulating influenza A and B viruses, were inoculated intranasally with 10^5^ to 10^7^ infectious units (PFU or EID_50_) of virus in a 1 mL volume. Ferrets were observed daily for clinical signs of infection; ferrets that lost >25% of preinoculation body weight or exhibited signs of neurological involvement were humanely euthanized. Nasal wash specimens were collected under anesthesia on alternate days 1 to 7 p.i. as described previously (40). Necropsy of virus-inoculated ferrets was conducted day 3 p.i. for collection and titration of nasal turbinates, trachea, and lung tissue as described previously (40, 41). Nasal wash and nasal turbinates titers are reported per milliliter, whereas trachea and lung tissue are reported per gram of tissue. All samples were immediately frozen at −70°C after collection and the viral titers were determined in either eggs or MDCK cells as specified. The limit of detection was 10^1.5^ EID_50_/mL or 10 PFU/mL. The growth or decay rates of ferret NW titer between a pair of measurement times, e.g., slope_1,3_ between day 1 and 3 p.i., were computed as the mean of the log_10_ NW titer measurements on day 3 minus that on day 1, divided by the time interval, e.g., 48 h.

### Standard time courses in Calu-3 cells.

The human bronchial epithelial cell line Calu-3 (ATCC) was employed for all *in vitro* experiments. Calu-3 cells were cultured on 24-mm or 12-mm semipermeable membrane inserts with a 0.4-μm pore size (Transwell, Corning) until transepithelial resistance >1,000 Ω·cm^2^ was achieved as described previously (18). Calu-3 cells were infected in at least triplicate with each virus at a multiplicity of infection (MOI) of 0.01 EID_50_ or PFU per cell and cultured at either 37°C or 33°C, as described previously (18). The ratio of apical media volume to surface area of the Transwell was generally the same in both insert sizes (4.67 cm^2^/2 mL and 1.12 cm^2^/0.5 mL for 6-well and 12-well plate formats, respectively). Cells were collected at 2, 24, 48, and 72 h p.i., frozen at −70°C, and subsequently titers of the viruses were determined in either eggs or MDCK cells as specified. Data were collected from previously published experiments or were generated for this study as indicated.

### Statistical and correlation analysis.

Peak log_10_ titers employed in all statistical analyses were determined for each well of Calu-3 cells or for each ferret individually and then averaged to yield mean log_10_ peak titer values (*n* ≥ 3 virus-infected Calu-3 wells or ferrets). As such, peak log_10_ titer averages may be inclusive of multiple time points or collection days. For tissues collected day 3 p.i. at necropsy, only viruses for which >50% of all inoculated ferrets had detectable infectious virus in each tissue were included in analyses (see Table S1 for tissue exclusion criteria). Pearson product-moment correlations were calculated to describe the relationship between *in vitro* and *in vivo* titer or weight loss data without adjustment for multiple comparisons. Statistical tests between categorical data (PB2, weight loss, lethality, transmission) were Pearson product-moment correlations. Predictive Power Scores were calculated using the R package ppsr v0.0.2 (42). All analyses were performed in R v4.0.3 (R Core Team 2020).

### Modeling analyses.

Modeling analyses are described in the Supplemental Methods.
