# Supplementary material for: Utility of Human In Vitro Data in Risk Assessments of Influenza A Virus Using the Ferret Model
Source: J Virol. 2023 Jan 5;97(1):e01536-22. doi: 10.1128/jvi.01536-22 (PMC9888249; doi:10.1128/jvi.01536-22)
Supplement: Supplemental file 1 — Supplemental methods, Fig. S1 to S5, and Table S1. Download jvi.01536-22-s0001.pdf, PDF file, 1.97 MB [file jvi.01536-22-s0001.pdf]

Supplemental Methods for: Utility of human *in vitro* data in risk assessment of influenza A virus using the ferret model

Hannah M. Creager, Troy J. Kieran, Hui Zeng, Xiangjie Sun, Joanna A. Pulit-Penaloza, Katie E. Holmes, Anders F. Johnson, Terrence M. Tumpey, Taronna R. Maines, Catherine A. A. Beauchemin, Jessica A. Belser

A Mathematical Methods

A.1 Mathematical model for influenza A virus infection kinetics

The *in vitro* infection of Calu-3 cells with different influenza A virus were simulated numerically using an age-structured, ordinary differential equation (ODE) model, introduced and described previously [4, 5]. The ODE MM, Eqn. (1), considers the infectious virus concentration, V, measured either on MDCK cells (PFU/mL) or in eggs (EID_{50}/mL), released by infected Calu-3 cells into the supernatant over the course of the *in vitro* infections.

$$
\begin{aligned}
\frac{\mathrm{d}T}{\mathrm{d}t} &= -\beta T V \\
\frac{\mathrm{d}E_1}{\mathrm{d}t} &= \beta T V - \frac{n_E}{\tau_E} E_1 \\
\frac{\mathrm{d}E_i}{\mathrm{d}t} &= \frac{n_E}{\tau_E} E_{i-1} - \frac{n_E}{\tau_E} E_i \quad \text{for } i = (2, \ldots, n_E) \\
\frac{\mathrm{d}I_1}{\mathrm{d}t} &= \frac{n_E}{\tau_E} E_{n_E} - \frac{n_I}{\tau_I} I_1 \\
\frac{\mathrm{d}I_j}{\mathrm{d}t} &= \frac{n_I}{\tau_I} I_{j-1} - \frac{n_I}{\tau_I} I_j \quad \text{for } j = (2, \ldots, n_I) \\
\frac{\mathrm{d}V}{\mathrm{d}t} &= \rho \sum_{j=1}^{n_I} I_j - \delta V
\end{aligned}
\tag{1}
$$

In this MM, a population of uninfected target cells (T) become infected by virus (V) at rate βV. The newly infected cells first enter the eclipse phase ($E_{i=1,\ldots,n_E}$) wherein they initiate intracellular virus replication. A time $\tau_E \pm (\tau_E/\sqrt{n_E})$ later, cells leave the eclipse phase to enter the infectious phase ($I_{j=1,\ldots,n_I}$) and begin releasing infectious virions (V), at constant rate ρ. A time $\tau_I \pm \tau_I/\sqrt{n_I}$ later, these infectious cells cease production and undergo apoptosis. The concentration of infectious virus in the supernatent decreases at rate δ as infectious virions lose infectivity over time.

From MM (1) parameters, one can compute the infecting time, t_{inf}, defined as the approximate time elapsed between the release of the very first virus progeny produced by a newly infected cell and the first latent infection (i.e. entry into E_1) of a secondary cell [3]. It is given by

$$
t_{\mathrm{inf}} = \sqrt{\frac{2}{\rho \beta}} \tag{2}
$$

and it has units of time, typically on the order of minutes or hours.

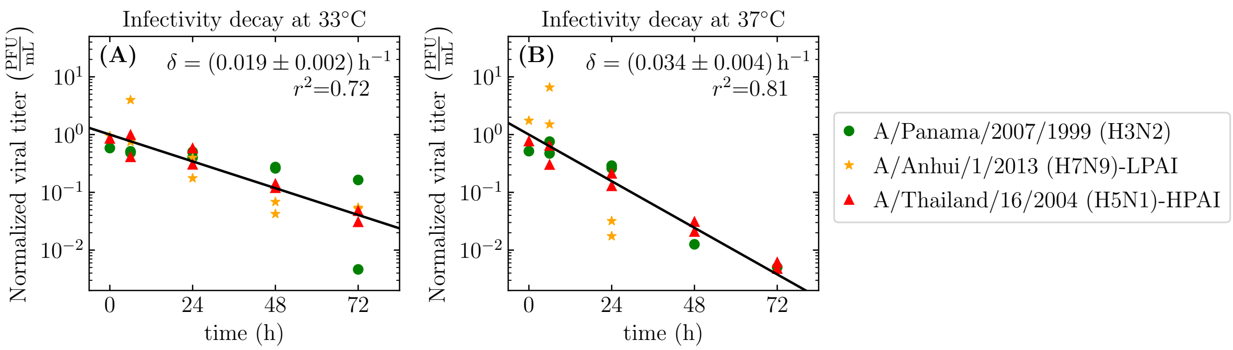

Figure A1: **Rate of loss of virions.** Virus stocks were incubated in the absence of any cells, but under the same conditions as the infection experiments, i.e. same medium at either 33°C or 37°C, to identify the rate at which infectious virions (PFU) lost infectivity. Virus concentration was quantified by plaque assay in MDCK cells, measured in duplicate from a single biological experiment, for each of the A/Panama/2007/1999 (H3N2), A/Anhui/1/2013 (H7N9), and A/Thailand/16/2004 (H5N1) influenza A virus strains. The decay rate and r^2 value for the log-lin linear regressions at each temperature are indicated in the upper left corner of each graph.

A.2 Parameter estimation from in vitro infections in Calu-3

MM (1) has a total of 7 parameters (β, τ_E, n_E, τ_I, n_I, ρ, δ) describing and controlling virus replication and the kinetics of infection spread. There are also initial conditions to consider. We assume all cells are initially uninfected ($T(0) = 1$, $E_i(0) = I_j(0) = 0\,\forall\, i, j$, corresponding to the fraction of cells in each state rather than their count), and we set $V(0) = V_0$ to be estimated along with the other MM parameters.

In MM (1), parameter δ corresponds to the rate at which infectious virions lose their infectivity. In order to estimate this quantity, we performed virus decay assays in which virions are incubated in the absence of cells under the same conditions as the infection experiments, i.e. in the same medium, at either 33°C or 37°C, and are titrated over time to evaluate the rate at which their infectivity is lost [4, 5]. Figure A1 shows that these rates were not virus-specific, as reported before [4]. We estimate $\delta = 0.02\ \mathrm{h}^{-1}$ at 33°C and $0.03\ \mathrm{h}^{-1}$ at 37°C. We use these values for all data sets across all strains in the parameter estimation below (Section B.2 herein evaluates the potential impact of this choice on our key findings).

In past work, and in preliminary analyses of the infection data in the present work, it was determined that the number of eclipse and infectious compartments or age-classes could be fixed to $n_E = n_I = 40$ without impacting other MM parameters or the quality of agreement between the MM and data [4, 6]. This leaves 5 MM parameters (β, τ_E, τ_I, ρ, V_0) to be estimated from the in vitro experimental infection data.

For each strain considered in the present work, MM (1) was used to simulate the experimentally measured viral titer over the course of the *in vitro* infections for a given 5-parameter set, $\vec{p} = \{\beta, \tau_E, \tau_I, \rho, V_0\}$. The posterior likelihood of a particular parameter set, following Bayes' theorem, is given by

$$\mathcal{P}_{\text{post}}(\vec{p}|\text{data}) = \frac{\mathcal{L}(\text{data}|\vec{p}) \cdot \mathcal{P}_{\text{prior}}(\vec{p})}{\mathcal{P}(\text{data})} \propto \mathcal{L}(\text{data}|\vec{p}) \cdot \mathcal{P}_{\text{prior}}(\vec{p}) \tag{3}$$

where we assume a uniform prior for τ_E and τ_I, and a log-uniform prior for ρ, β, V_0, such that

$$\mathcal{P}_{\text{prior}}(\vec{p}) = \frac{1}{\beta \cdot \rho \cdot V_0} \tag{4}$$

and we assume the variability in the viral titer measurements, $V(t)$, follows a log-normal distribution, such

that

$$\mathcal{L}(\text{data}|\vec{p}) = \exp\left[-\frac{\text{SSR}(\vec{p})}{2\sigma^2}\right] = \exp\left[-\sum_{i=1}^{N_\text{pts}} \frac{\left\{\log_{10}\left[V_i^\text{model}(\vec{p})\right] - \log_{10}\left[V_i^\text{data}\right]\right\}^2}{2\sigma^2}\right] \tag{5}$$

where V_i^data is the ith experimentally measured virus time point, and $V_i^\text{model}(\vec{p})$ is its corresponding MM-predicted value given parameter set $\vec{p}$. We use $\sigma = 1.0$, i.e. we assume that the intra-experimental variability in $\log_{10}(V)$ between the 3 replicates performed for each infection has a typical standard deviation of $\sim \pm 1$. Since virus decay was not yet visible in most data sets by the last measurement time point at 72 h, there was often little to no constraint imposed on the upper bound value of the infectious cell lifespan (τ_I). We therefore constrained τ_I to be at most 5 days. The eclipse phase duration is best constrained by single cycle infections where all cells are infected at once. Since all infections herein proceeded over multiple infection cycles, τ_E was constrained to values between 30 min and 18 h in order to avoid unrealistic values.

A Markov chain Monte Carlo (MCMC) approach, implemented by the `phymcmc` python module [1] which itself makes use of the `emcee` python module [2], was used to estimate the posterior likelihood distributions (PostPLDs) of the 5-parameter $\vec{p}$. The PostPLDs for each virus is the result of 100 chains of 10,000 steps each (1,000,000 parameter sets), after a burn-in of 10,000 steps. This burn-in was confirmed to be sufficient for the chains to have reached convergence, i.e. for the walkers' distribution (median and 1σ value of all walker's accepted parameters, computed at each step) to have become constant (more or less flat) over the 10,000 steps, visualized using `phymcmc`'s diagnostic plots.

A.3 MM-simulated infection time course and MM-derived quantities in Calu-3

The 1,000,000 parameter sets estimated as described herein in Section A.2 were sampled at random with replacement to produce 10,000 MM-simulated infection time courses. From these time courses and the associated parameters, additional MM-derived quantities were obtained. The quantities considered were:

maxlog$_{10}$V : the maximum value of the $\log_{10}$ titer over the MM-simulated time course which is actually 0–86 h. It is contaminated by the virus measurement units.

meanlog$_{10}$V : the mean value of $\log_{10}[V(t)]$ over the entire duration of the MM-simulated infection (0–86 h). It is equivalent to the AUC. It is contaminated by the virus measurement units.

t_inf : the infecting time, i.e. the average time it will take for one infected cell to infect another, computed from the MM parameters using Eqn. (2). It is not contaminated by the virus measurement units.

growth (units of per hour) : the exponential growth rate of the infection estimated from the MM-predicted titer time course. It depends on nearly all of the MM parameters so it should provide a robust, summarizing measure of all the MM parameters. It is not contaminated by the virus measurement units.

$t_{1\%\max V}$, $t_{\text{meanlog}_{10}V}$ **(units of hour)** : time to reach 1% of the MM-predicted peak titer, and time to reach meanlog$_{10}V$, respectively. Neither are contaminated by the virus measurement units.

A.4 Effect of temperature on MM-derived quantities in Calu-3 infections

MM-predicted viral titer time courses for the 36 viruses for which in vitro infections in Calu-3 were conducted at 2 different temperatures are shown in Figure A2. While peak virus titer was typically unaffected by temperatures, the exponential titer growth rate (referred to as growth) was larger, and the time at which the virus titer reaches 1% of its MM-predicted peak value (vertical solid lines) occurred sooner at 37°C than at 33°C.

There were, of course, some exceptions: infections with A/New York/107/2003, A/Shoveler/Egypt/00215-NAMRU3/2007, and A/chicken/Texas/18-007912-2/2018 yielded lower peak viral titer at 33°C than at 37°C.

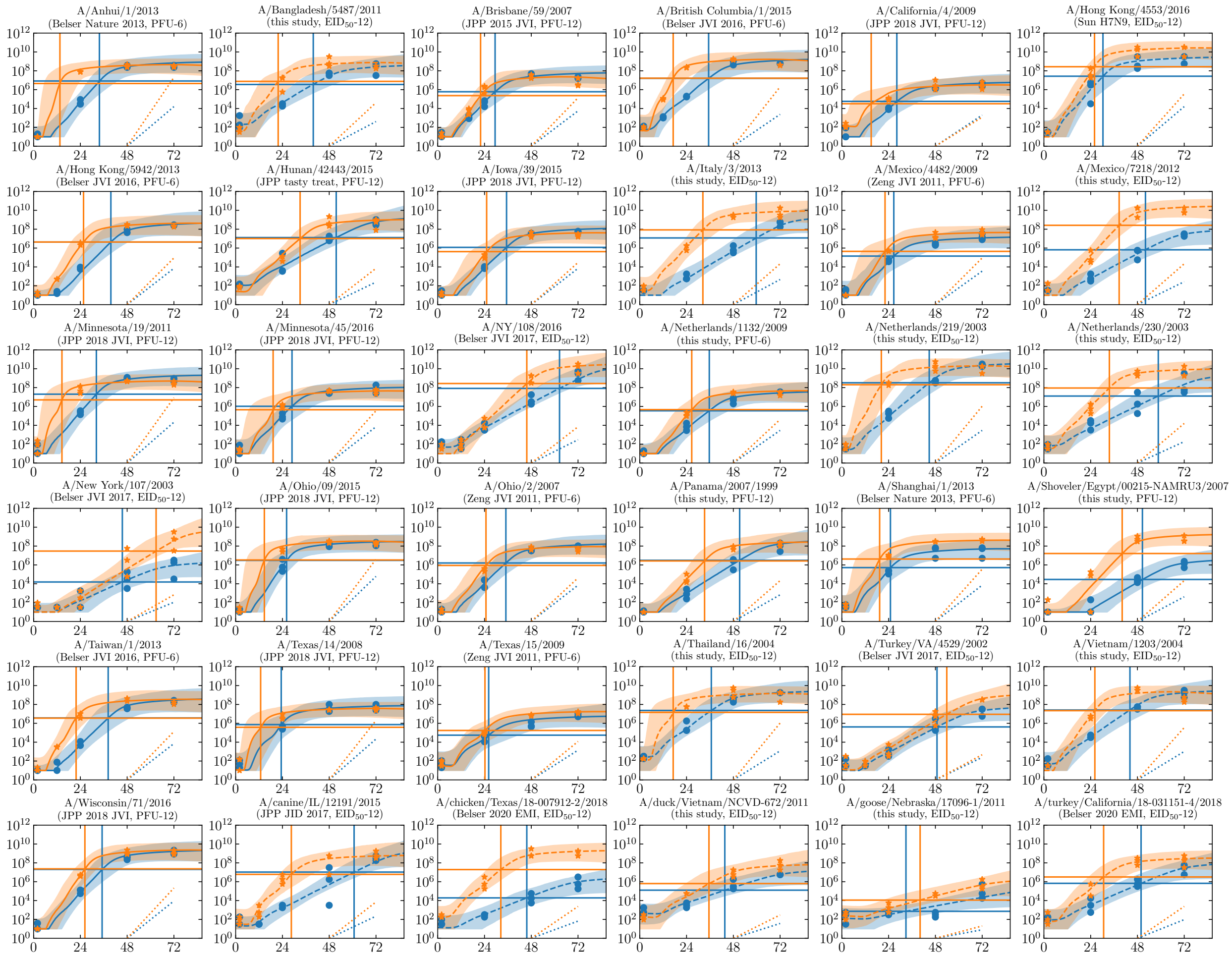

Figure A2: **Time course of experimental and MM-simulated in vitro influenza A virus infections in Calu-3.** Calu-3 were infected with different influenza A virus strains. For each strain, infection was performed in triplicate, and for the strains shown here, infection was performed at either 33°C (blue, circle) or 37°C (orange, star). The experimental measurements are shown as symbols (circle or star), the MM-predicted viral titer time course for the parameter set consisting of each parameter's median value (solid line) is shown along with the bounds of the 95-percentile time course at each time point (shaded regions). The horizontal and vertical solid lines represent 1% of the MM-predicted peak viral titer and the time at which it occurs, respectively. The dashed diagonal lines in the bottom right of each graph is a visual representation of the exponential growth rate of the virus (slope).

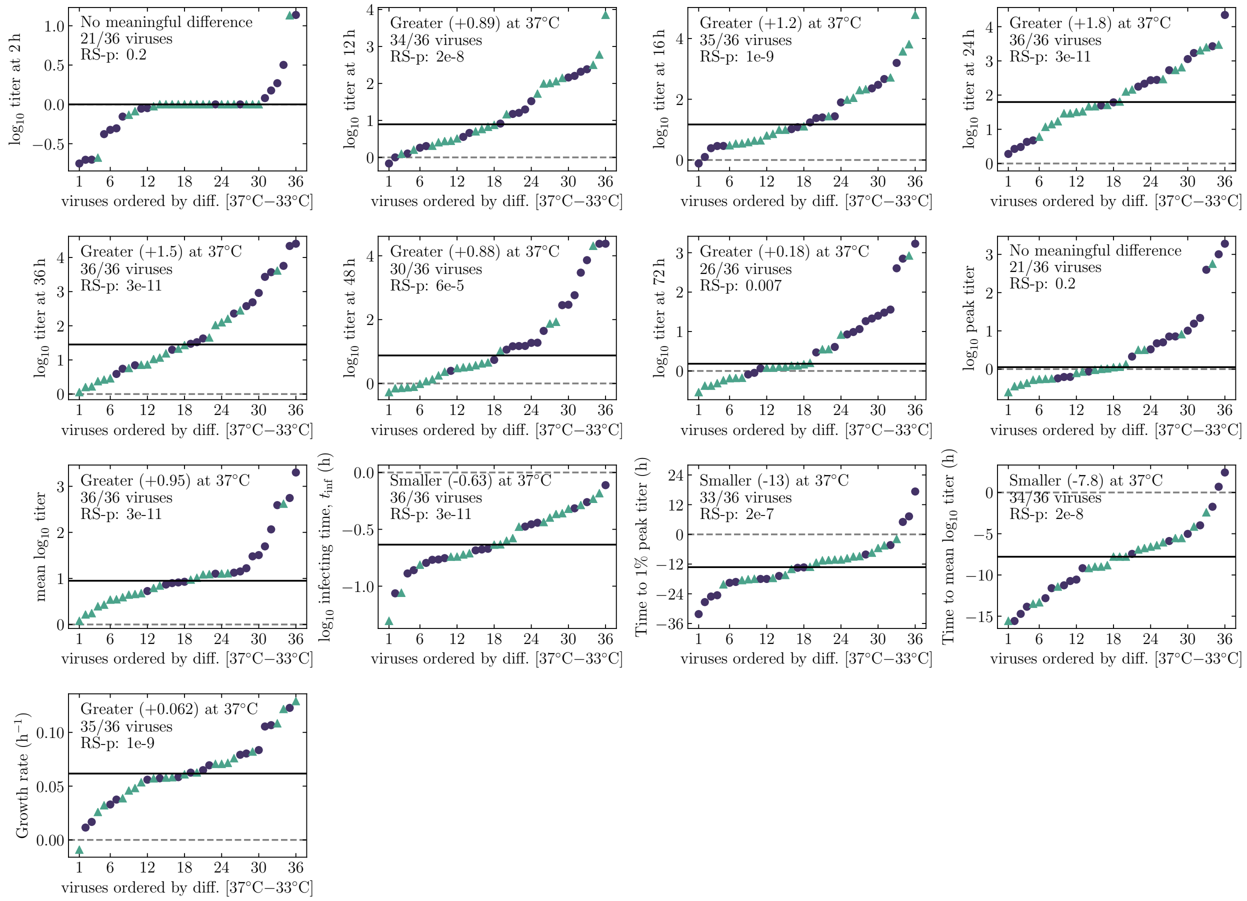

Figure A3: **Comparing the effect of temperatures on MM-derived quantities.** Difference in MM-derived quantity at 37°C minus that at 33°C, computed for all viruses ($n = 36$) of Figure A2 titered in eggs (circle) or cells (triangles), ordered from smallest (or most negative) to largest (or most positive), i.e. viruses are not in the same order in each panel. The horizontal dashed line corresponds to zero (no difference), and the horizontal solid line corresponds to the median difference over the 36 viruses, also indicated in parenthesis (e.g., 'Greater (+1.8)').

For the infection with A/New York/107/2003, we see that the reduced peak titer for infections at 33°C (one measure of interest) was such that its viral titer curve reached 1% of its peak titer faster than the infection at 37°C. This shows that any one single measure of a dynamical infection curve with multiple features can yield confusing results. It is such confounders that can lead to lower correlations between in vitro and in vivo measures, and this could occur even if, in reality, there was a good correspondence between the overall shape of the infection titer curves in vitro and in vivo.

Figure A3 shows quantitatively the effect of temperatures on MM-derived quantities, ordered from most to least significant effect. Like Figure A2, it shows the higher titer growth rate (parameter 'growth') and higher titers measured during the growth phase (at 24 h and 36 h), and the shorter time to reach various intermediate at higher temperature. For each measure, we report the median difference, and the fraction of viruses in which this difference was observed. For example, the median difference between the MM-predicted $\log_{10}$ titer at 24 h at 37°C minus that at 33°C was +1.8. This means that 50% of the titers measured at 24 h were at least 63× ($10^{1.8}$) higher in infections conducted at 37°C than at 33°C. The additional numbers

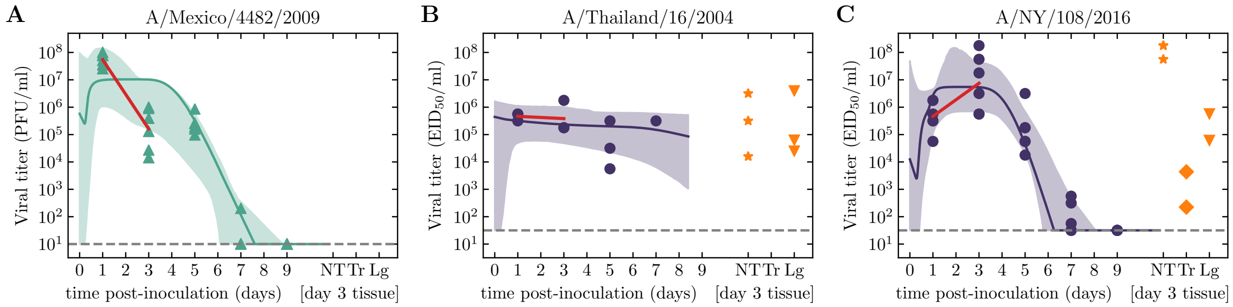

Figure A4: **Example time course of experimental and MM-simulated in vivo influenza A virus infections in ferrets.** Nasal wash (NW) viral titers in at least 3 ferrets infected with the same influenza A virus strains were measured every other day, starting on the first day post-inoculation up to 9 days p.i.. The experimental NW measurements titrated either in eggs (EID_{50}, circle) or MDCK cells (PFU, triangle) are shown alongside the MM-predicted median (solid line) and 95-percentile infection time course (shaded regions). For some strains (B,C), tissue titers at day 3 p.i. were also obtained from the nasal turbinates (NT, star), trachea (Tr, diamond), and lung (Lg, down triangle). While the MM-predicted initial viral titer and thus the initial growth kinetics is not constrained by the available titer data, a simple linear regression of the median $\log_{10}$ titer difference between two measureemnt time points ($\text{slope}_{1,3}$, red linear segment) can help discriminate between infections that peak and resolve early, show sustained growth, or grow and peak later.

(e.g., 36/36 viruses, RS-p: 10^{-11}) mean that this difference was positive in all 36 viruses measured, and

$$\text{RS-p} = 2 \times \sum_{k=s}^{n} \text{Binomial}(k|n=36, p=0.5) = 2 \times \sum_{k=s}^{n} \frac{n!}{k!(n-k)!} p^k (1-p)^k \tag{6}$$

where $n = 36$ is the number of viruses tested, $p = 0.5$ is the null-hypothesis (i.e., if the quantity is neither greater nor smaller, it should be 50:50 greater:smaller), and s is the number of viruses for which the difference was positive. Here s is +1 for every virus for which the difference was positive, and +0.5 when the difference was zero. The $\sum_{k=s}^{n}$ gives the likelihood that s or more of the 36 differences would be positive, and the $2\times$ accounts for the two-tailed test, i.e. testing both the assumption that the quantity is greater or smaller due to temperature.

A.5 MM analysis of infections in ferrets and its limitations

The ODE MM (1) used herein to analyze infections in Calu-3 cells has also routinely been applied to analyze *in vivo* virus infections. Unfortunately, the experimentally measured NW titers considered in the present study lack critical information about the initial kinetics of infection establishment and expansion within the host, which is required to constrain the MM parameters, or even the MM-predicted infection time course.

Figure A4 presents MM-estimated time courses for 3 strains. It illustrates well how this lack of information translates to large uncertainties in the MM-predicted titer curves. Notably, whereas the information provided by the data for infections in Calu-3 results in relatively smooth and tightly constrained MM-predicted 95-percentile initial dose (virus at time zero) and time course (Figure A2), the same analysis in ferret critically lack a well-identified initial dose and up-slope. The MM analysis shows that the viral titer at time zero could be almost anything. This is in part because, without further data or assumptions, the early infection kinetics could be the result of a high initial dose which is responsible for most of the infection with minimal infection spread beyond that resulting from the initial inoculum (titer at time zero is equal to the titer at 1 day p.i. with no titer growth, i.e. flat line, between day 0 and 1), and it could equally be the result of a low initial dose (barely above or even below the detection limit) followed by very rapid and widespread growth

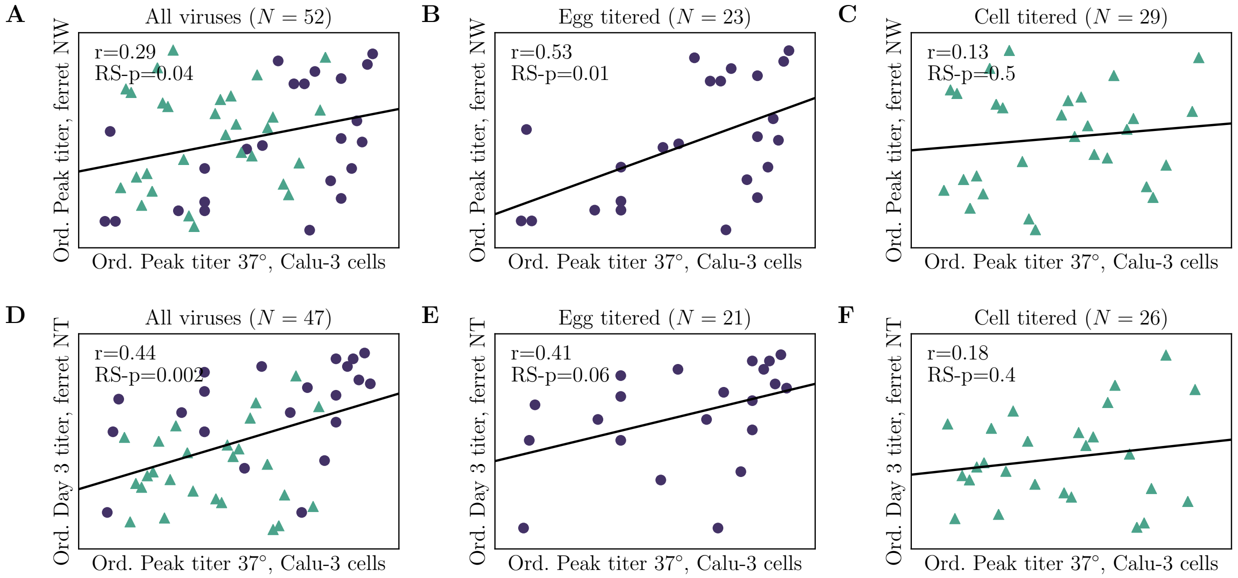

Figure B5: **Re-analysis of Figure 1 using ordinal rank.** The x and y axes and point coordinates represent the ordinal (Ord.) rank of each measure rather than the measure itself. For measures with equal rank, the averaged rank of the equal measures was used. The Pearson correlation coefficient and RS-p were then computed on the measure's ordinal rank shown.

(10,000-fold increase in titer between day 0 and 1 p.i.). While all ferrets were inoculated with a known viral titer dose, we cannot make use of this information in the MM analysis since there could be large discrepancies between the dose administered and the dose received (i.e. how much of the dose caused successful infection), and notably these discrepancies can reasonably be expected to vary for different strains due to factors such as host tropism.

Irrespective of the underlying kinetics of the early infection establishment, it is possible that similar mechanisms and kinetics are in play among infections where the NW titer has already peaked by day 1 p.i., when compared to those infections with sustained titers (little to no change between day 1 and 3 p.i.), and those where the infection is still spreading and expanding between days 1 and 3 p.i.. For this reason, we considered a simpler analysis to quantify and document the growth or decay rates of ferret NW titer between pair of measurement time points, such as $\text{slope}_{1,3}$ which corresponds to the difference in the mean $\log_{10}$ NW titer between day 3 and day 1 p.i. divided by the time interval (48 h).

B Approach validations

B.1 Pearson vs rank correlation coefficients

Pearson correlation coefficients, denoted by r, were used throughout the work to quantify the extent to which measures in Calu-3 relate to those in ferret. Figure B5 reproduces the analysis presented in Figure 1A–1F of the manuscript. It shows that the rank (RS-p) and strength (r) of the correlations remain mostly unchanged whether the analysis is performed on the measures themselves or on the ordinal rank of these same measures.

B.2 Fixed vs free rate of infectious virus loss, δ

The analysis presented in the main text is based on MM parameter estimation where the virus decay rate (δ) was fixed based on temperature, as explained herein above. In Figure B6, we explore the robustness of

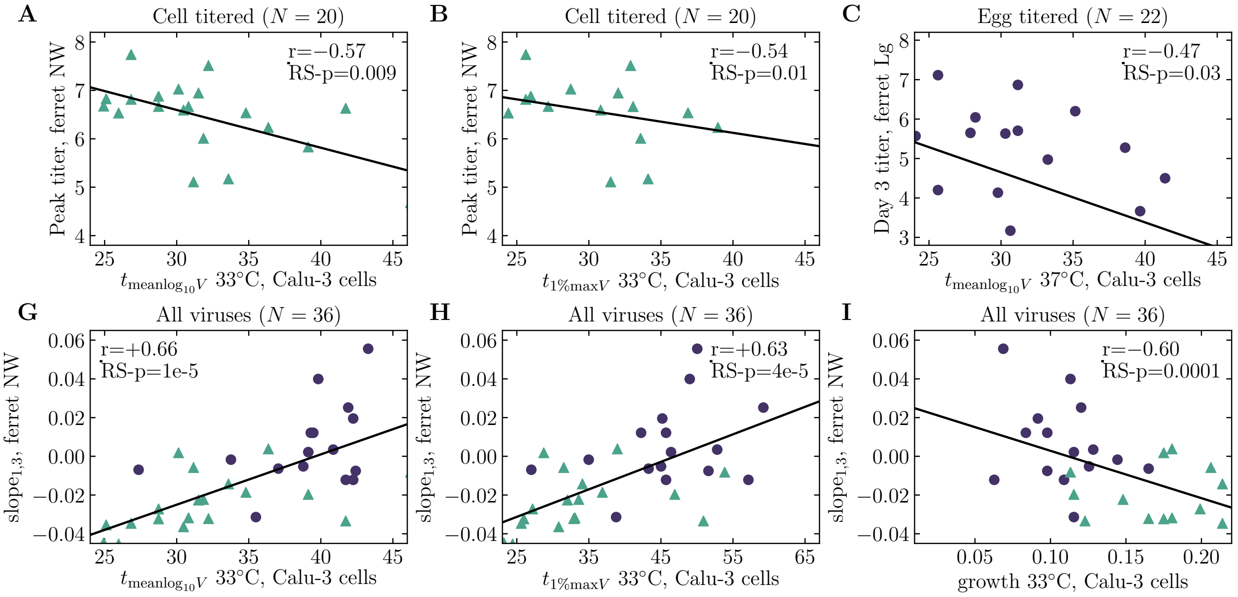

Figure B6: **Re-analysis of Figure 3 using a free virus loss rate δ.** The analysis presented in Figure 3 panels A,B,C and G,H,I in the main text was repeated for all strains for a set of MM parameters wherein the virus decay rate δ was free to vary $\in [0.001, 1.0]$ h^{-1}. The results mostly remain unchanged from those presented in the main text.

the findings presented in the main text as we relax the constraint on the virus decay rate and allow it to vary freely $\in [0.001, 1.0]$ h^{-1}. It shows that the rank (RS-p) and strength (r) of the correlations seem largely robust to the specific value of the virus decay rate, δ. This is not surprising because our correlation analyses focus on MM-extracted bulk features of the measured viral titers, e.g. viral titer peak, growth rate, time to reach some titer milestone, rather than on MM replication parameters, e.g. virus production (ρ) or cell entry (β) rate. This choice was made specifically because the former are well constrained and informed by the available data whereas the latter are not.

References

[1] C. A. A. Beauchemin. *phymcmc: A convenient wrapper for emcee*, 2019.

[2] D. Foreman-Mackey, D. W. Hogg, D. Lang, and J. Goodman. emcee: The MCMC hammer. *Publ. Astron. Soc. Pac.*, 125(925):306–312, March 2013. URL: https://github.com/dfm/emcee, arXiv:1202.3665, doi:10.1086/670067.

[3] B. P. Holder, P. Simon, L. E. Liao, Y. Abed, X. Bouhy, C. A. A. Beauchemin, and G. Boivin. Assessing the in vitro fitness of an oseltamivir-resistant seasonal A/H1N1 influenza strain using a mathematical model. *PLoS ONE*, 6(3):e14767, 24 March 2011. doi:10.1371/journal.pone.0014767.

[4] E. G. Paradis, L. T. Pinilla, B. P. Holder, Y. Abed, G. Boivin, and C. A. A. Beauchemin. Impact of the H275Y and I223V mutations in the neuraminidase of the 2009 pandemic influenza virus in vitro and evaluating experimental reproducibility. *PLoS ONE*, 10(5):e0126115, 20 May 2015. doi:10.1371/journal.pone.0126115.

[5] L. T. Pinilla, B. P. Holder, Y. Abed, G. Boivin, and C. A. A. Beauchemin. The H275Y neuraminidase mutation of the pandemic A/H1N1 virus lengthens the eclipse phase and reduces viral output of infected

cells, potentially compromising fitness in ferrets. *J. Virol.*, 86(19):10651–10660, October 2012. doi:10.1128/JVI.07244-11.

[6] P. F. Simon, M.-A. de La Vega, E. Paradis, E. Mendoza, K. M. Coombs, D. Kobasa, and C. A. A. Beauchemin. Avian influenza viruses that cause highly virulent infections in humans exhibit distinct replicative properties in contrast to human H1N1 viruses. *Sci. Rep.*, 6:24154, 15 April 2016. doi:10.1038/srep24154.

Supplemental Figures.

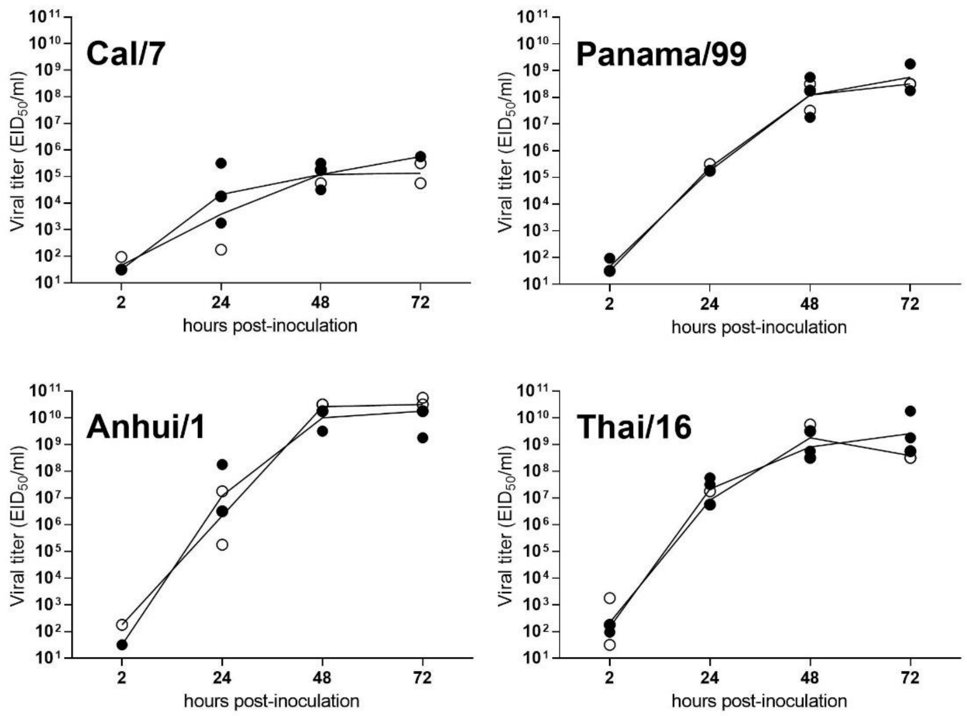

Supplemental Figure 1. Evaluation of Transwell insert size on IAV replication in Calu-3 cells. Calu-3 cells were grown to confluence in either 12mm (closed circles) or 24 mm (open circles). Transwell inserts and inoculated in duplicate or triplicate at a MOI of 0.01 with the representative IAV shown (Cal/7, A/California/7/2009 pdm09 H1N1; Panama/99, A/Panama/2007/1999 seasonal H3N2; Anhui/1, A/Anhui/1/2013 LPAI H7N9; Thai/16, A/Thailand/16/2004 HPAI H5N1). Cells were cultured post-inoculation at 37°C and supernatant was collected at 2, 24, 48, and 72 hours p.i. for subsequent titration in embryonated chicken eggs for determination of infectious titer (reported as $\log_{10}$ EID_{50}/ml). Limit of detection was $10^{1.5}$ EID_{50}/ml. Individual well titers are shown with solid line representing mean at each timepoint.

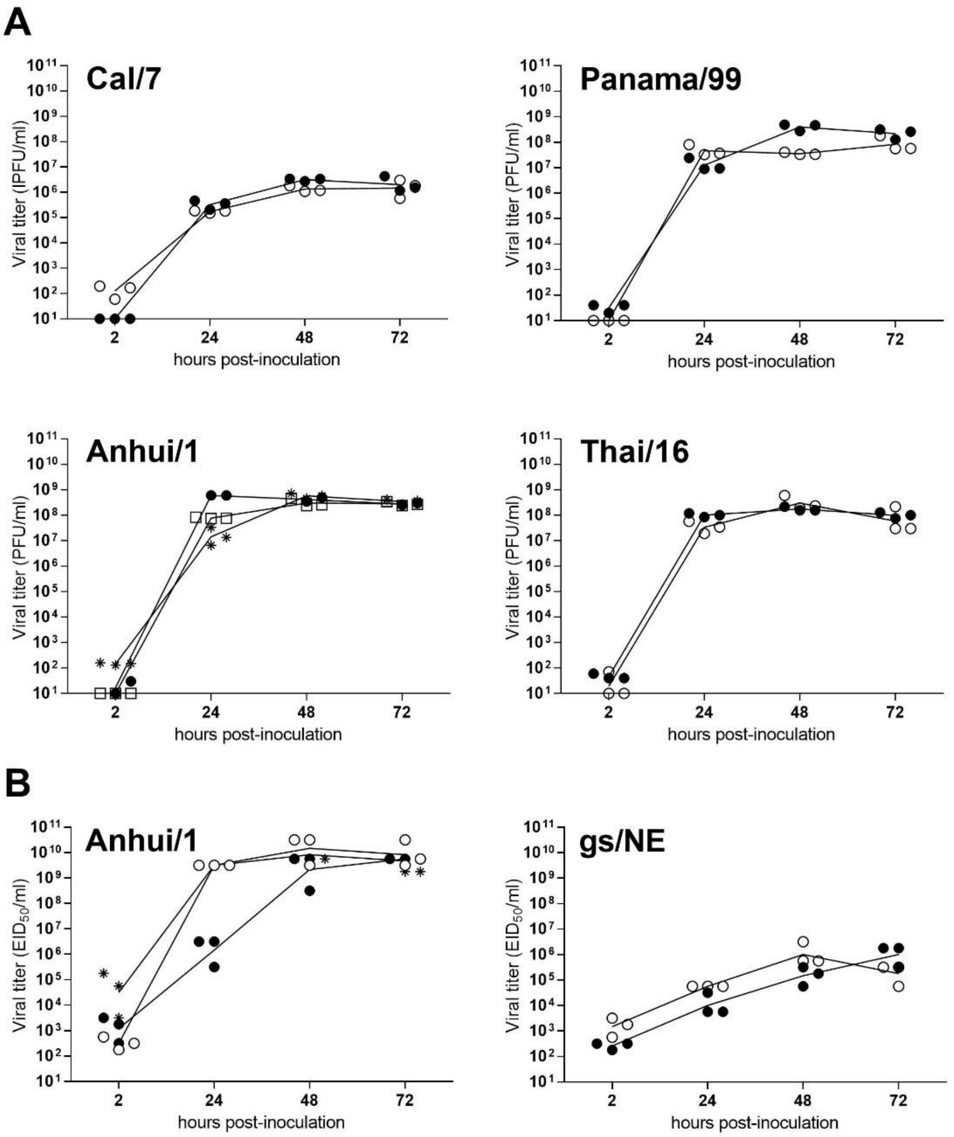

Supplemental Figure 2. Reproducibility of independently conducted IAV replication kinetics in Calu-3 cells. Calu-3 cells were grown to confluence in either 12 mm or 24 mm Transwell inserts and inoculated in triplicate at a MOI of 0.01 with the representative IAV shown (Cal/7, A/California/7/2009 pdm09 H1N1; Panama/99, A/Panama/2007/1999 seasonal H3N2; Anhui/1, A/Anhui/1/2013 LPAI H7N9; Thai/16, A/Thailand/16/2004 HPAI H5N1; gs/NE, A/goose/Nebraska/17096-1/2011 LPAI H7N9). Cells were cultured post-inoculation at 37°C and supernatant was collected at 2, 24, 48, and 72 hours p.i. for subsequent titration in MDCK cells (A) or embryonated chicken eggs (B) for determination of infectious titer (reported as log_{10} PFU/ml or EID_{50}/ml, respectively). Shapes represent independent timecourse experiments (titrated in eggs or cells as specified in Table 1); solid line represents mean at each timepoint. Limit of detection was 10^1 PFU or $10^{1.5}$ EID_{50}/ml.

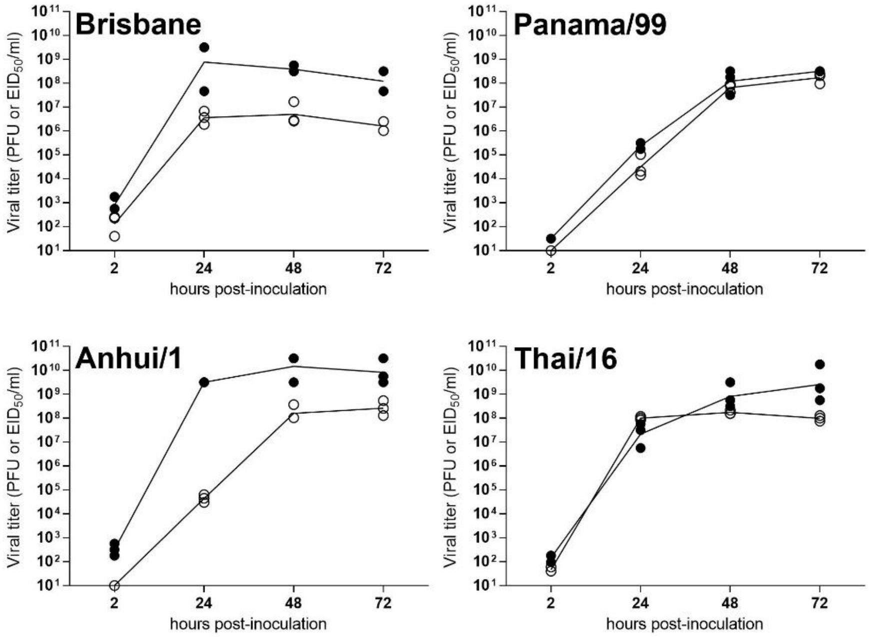

Supplemental Figure 3. Dependence of timecourse viral titer on titration matrix employed. Calu-3 cells were grown to confluence in 12mm transwell inserts and inoculated in triplicate at a MOI=0.01 with the representative IAV shown (Brisbane, A/Brisbane/57/2007 seasonal H1N1; Panama/99, A/Panama/2007/1999 seasonal H3N2; Anhui/1, A/Anhui/1/2013 LPAI H7N9; Thai/16, A/Thailand/16/2004 HPAI H5N1). Cells were cultured post-inoculation at 37°C and supernatant was collected at 2, 24, 48, and 72 hours p.i. for subsequent titration in embryonated chicken eggs (closed circles) or MDCK cells (open circles) for determination of EID_{50} or PFU titer, respectively. Limit of detection was 10^1 PFU or $10^{1.5}$ EID_{50}/ml. Individual well titers are shown with solid line representing mean at each timepoint.

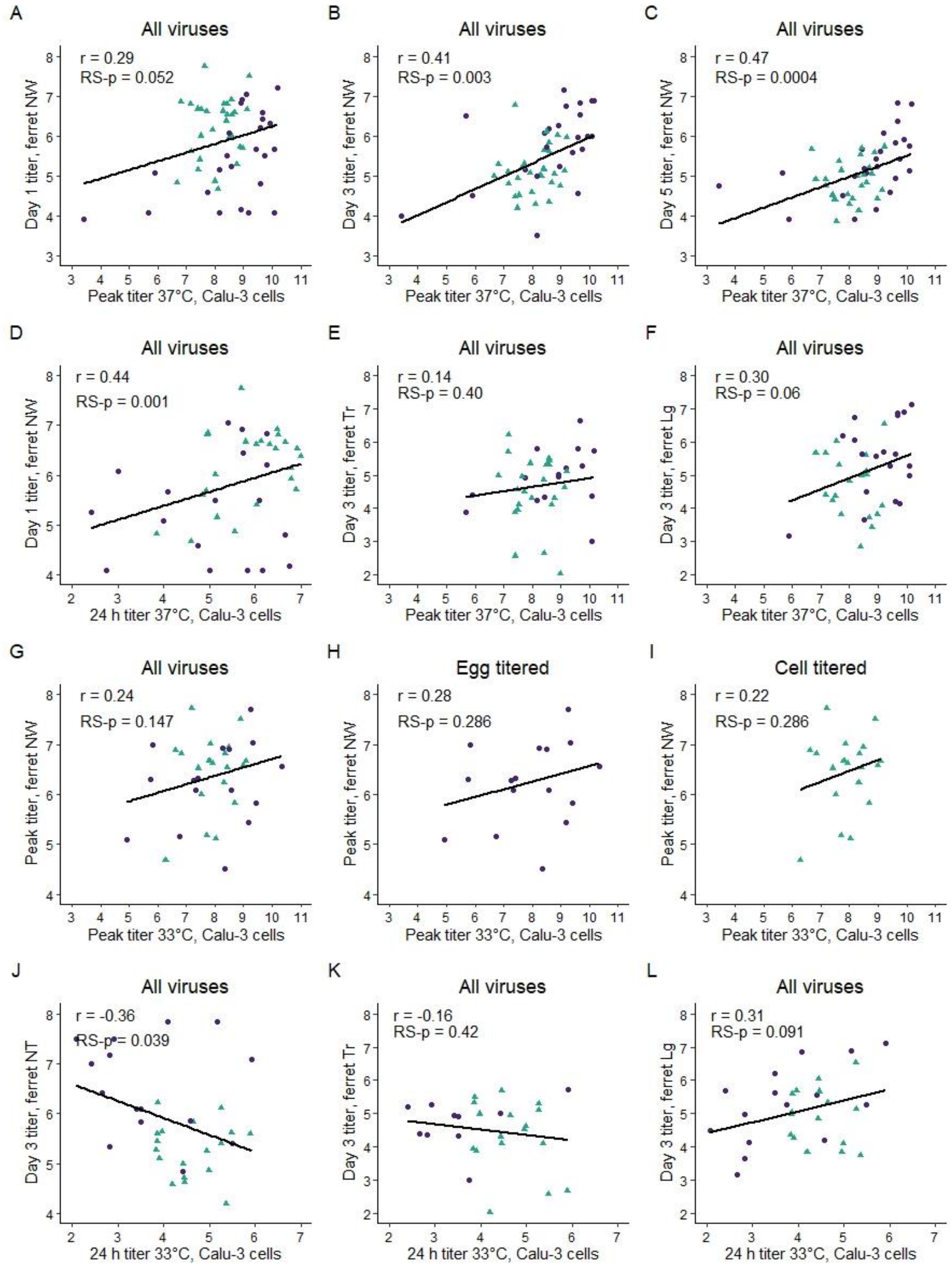

Supplemental Figure 4. Correlations between ferret nasal wash, ferret nasal turbinate, and Calu-3 viral titers following IAV infection. Best fit line determined by least squares regression; r values are from Pearson correlation tests (see Supplemental Table 2). In mixed datasets, sample titration matrix is identified as eggs (circles) or cells (triangles) as specified in Table 1. Day 1 (A), 3 (B), and 5 (C) p.i. NW vs peak Calu-3 titer (37°C) for all viruses in dataset. Day 1 p.i. NW vs 24 h Calu-3 titer (37°C) (D). Day 3 p.i. Tr (E) and Lg (F) titer vs peak Calu-3 titer (37°C). Peak ferret NW vs peak Calu-3 titer (33°C) for all viruses in dataset (G), viruses titered in eggs only (H), or viruses titered in cells only (I). Day 3 p.i. NT (J), Tr (K), and Lg (L) titer vs 24 h Calu-3 titer (33°C). Units for viral titers shown are log_{10} PFU or EID_{50}/ml or g.

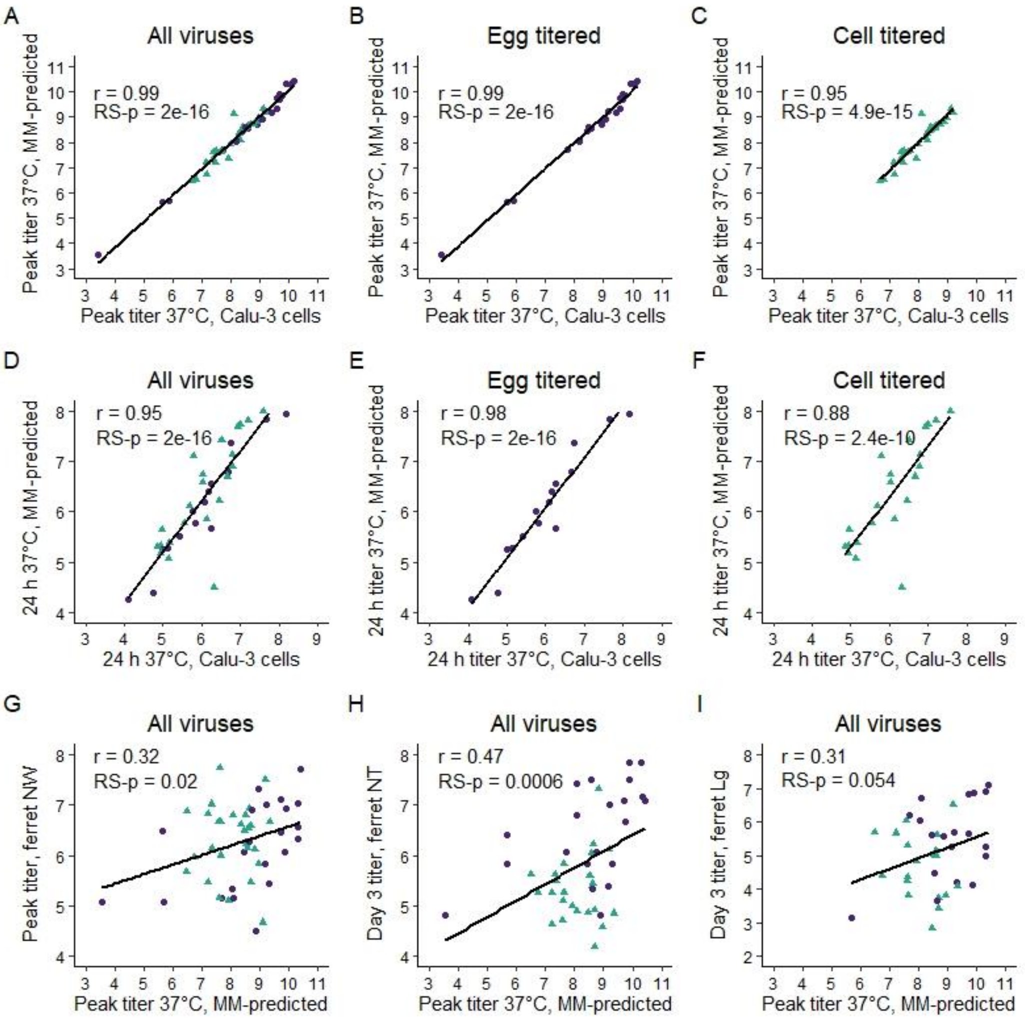

Supplemental Figure 5. Correlations between Calu-3 and ferret viral titers following IAV infection with MM-predicted values. Best fit line determined by least squares regression; r values are from Pearson correlation tests (see Supplemental Table 3A). In mixed datasets, sample titration matrix is identified as eggs (circles) or cells (triangles) as specified in Table 1. Peak Calu-3 titer (37°C) vs MM-predicted peak titer for all viruses in dataset (A), viruses titered in eggs only (B), or viruses titered in cells only (C). 24 h Calu-3 titer (37°C) vs MM-predicted 24 h titer for all viruses in dataset (D), viruses titered in eggs only (E), or viruses titered in cells only (F). MM-predicted peak titer vs peak ferret NW (G), day 3 p.i. NT (H), or day 3 Lg (I). Units for viral titers shown are $\log_{10}$ PFU or EID_{50}/ml or g.

Supplemental Table 1. Supporting in vitro and in vivo data for influenza A viruses used in this study.

Virus Name	Subtype[a]	Titration units[b]	Peak Calu-3[c]	Peak NW[d]	NT[e]	Tr[e]	Lg[e]
A/Brisbane/59/2007	H1N1	PFU	7.40	7.02	pos	pos	neg
A/California/4/2009	H1N1pdm09	PFU	6.82	6.88	pos	pos	pos
A/Mexico/4482/2009	H1N1pdm09	PFU	7.64	7.74	pos	pos	pos
A/Texas/15/2009	H1N1pdm09	PFU	7.16	6.82	pos	pos	pos
A/Netherlands/1132/2009	H1N1pdm09	PFU	7.50	6.01	pos	pos	pos
A/Ohio/2/2007	H1N1v	PFU	7.99	5.11	pos	pos	pos
A/Texas/14/2008	H1N1v	PFU	7.53	6.67	pos	pos	pos
A/Iowa/39/2015	H1N1v	PFU	7.41	5.17	pos	pos	pos
A/Ohio/9/2015	H1N1v	PFU	8.42	6.81	pos	pos	
A/Hunan/42443/2015	H1N1v	PFU	8.97	5.83	pos	pos	pos
A/Minnesota/19/2011	H1N2v	PFU	8.69	6.59	pos	pos	pos
A/Minnesota/45/2016	H1N2v	PFU	7.72	6.67	pos	pos	pos
A/Wisconsin/71/2016	H1N2v	PFU	9.13	6.66	pos	pos	pos
A/Panama/2007/1999	H3N2	PFU	8.28	6.63	NA	NA	NA
A/Panama/2007/1999	H3N2	EID_{50}	8.95	6.92	NA	NA	NA
A/Perth/16/2009	H3N2	PFU	6.67	5.68	NA	NA	NA
A/canine/Illinois/12191/2015	H3N2 canine	EID_{50}	8.92	6.92	pos	pos	
A/Kansas/13/2009	H3N2v	PFU	8.77	5.46	pos	pos	pos
A/Minnesota/11/2010	H3N2v	PFU	8.88	6.12	pos	neg	neg
A/Pennsylvania/14/2010	H3N2v	PFU	7.93	6.14	pos	pos	
A/Indiana/8/2011	H3N2v	PFU	8.32	6.81	pos	neg	neg
A/Iowa/8/2011	H3N2v	PFU	8.59	6.16	pos	pos	pos
A/Ohio/13/2012	H3N2v	PFU	8.39	6.48	pos	pos	pos
A/Michigan/39/2015	H3N2v	PFU	7.47	5.47	pos	pos	neg
A/Ohio/27/2016	H3N2v	PFU	7.17	5.96	pos	pos	pos
A/Thailand/16/2004	H5N1	EID_{50}	9.42	5.83	pos	NA	pos
A/Vietnam/1203/2004	H5N1	EID_{50}	9.58	5.44	pos	NA	pos
A/Bangladesh/5487/2011	H5N1	EID_{50}	8.92	4.5	pos	pos	pos
A/duck/Vietnam/NCVD-672/2011	H5N1	EID_{50}	7.75	5.17	pos	pos	pos
A/chicken/Texas/18-007912-2/2018	H7N1	EID_{50}	9.17	7.0	pos	pos	pos
A/turkey/Virginia/4529/2002	H7N2	EID_{50}	8.5	6.28	pos	NA	pos
A/New York/107/2003	H7N2	EID_{50}	8.58	6.29	pos	NA	pos
A/New York/108/2016	H7N2	EID_{50}	10.08	7.04	pos	pos	pos
A/Canada/504/2004	H7N3	EID_{50}	9.08	7.33	NA	NA	NA
A/Mexico/7218/2012	H7N3	EID_{50}	10.08	6.33	pos	pos	pos
A/turkey/California/18-031151-4/2018	H7N3	EID_{50}	8.42	6.08	pos	pos	pos
A/Netherlands/219/2003	H7N7	EID_{50}	9.92	6.56	pos	NA	pos
A/Netherlands/230/2003	H7N7	EID_{50}	9.67	6.94	pos	NA	pos
A/Italy/3/2013	H7N7	EID_{50}	9.75	6.08	pos	pos	pos
A/turkey/Indiana/1403/2016	H7N8	EID_{50}	8.17	5.17	pos	pos	pos
A/turkey/Indiana/1573-2/2016	H7N8	EID_{50}	8.17	5.33	pos	pos	pos
A/goose/Nebraska/17096-1/2011	H7N9	EID_{50}	5.89	5.08	pos	pos	pos
A/chicken/Tennessee/17-007147-2/2017	H7N9	EID_{50}	3.42	5.08	pos	neg	neg
A/chicken/Tennessee/17-007431-3/2017	H7N9	EID_{50}	5.67	6.50	pos	pos	neg
A/shoveler/Egypt/00215-NAMRU3/2007	H7N9	PFU	8.11	4.68	NA	NA	NA
A/Anhui/1/2013	H7N9 (1)	PFU	8.58	6.94	pos	pos	pos
A/Shanghai/1/2013	H7N9 (1)	PFU	8.59	6.53	pos	pos	pos
A/Taiwan/1/2013	H7N9 (1)	PFU	8.49	6.23	pos	pos	pos
A/Hong Kong/5942/2013	H7N9 (2)	PFU	8.43	6.53	pos		pos
A/British Columbia/1/2015	H7N9 (3)	PFU	9.21	7.51	pos	pos	pos
A/Hong Kong/4553/2016	H7N9 (5)	EID_{50}	10.17	7.71	pos	pos	pos
A/Guangdong/17SF003/2016	H7N9 (5)	EID_{50}	9.67	7.13	pos	pos	pos
A/Taiwan/1/2017	H7N9 (5)	EID_{50}	9.58	6.46	pos	pos	pos

[a]Epidemiological wave from which H7N9 viruses were isolated from humans is indicated in parentheses. v, denotes variant virus. Virus descriptions are presented in Table 1. [b]The method of titration for detection of infectious virus. EID_{50}, 50% egg

infectious dose; PFU, plaque forming units in London-line Madin Darby Canine Kidney (MDCK) cells. [c]Mean peak $\log_{10}$ PFU or EID_{50}/ml titer from n≥3 independent wells of Calu-3 cells infected at an MOI=0.01 and cultured at 37°C. [d]Mean peak $\log_{10}$ PFU or EID_{50}/ml titer from n≥3 ferrets inoculated intranasally with 10^5-10^7 infectious units of virus. [e]pos, indicates >50% of tissues collected day 3 p.i. possessed a detectable infectious virus and the mean titer of these tissues with positive virus detection are included in analyses presented in the main text. NA, indicates tissue was not collected during necropsy. Neg, indicates that no infectious virus was detected in this tissue above the limit of detection (10^1 PFU/ml, $10^{1.5}$ EID_{50}/ml). No mark, indicates <50% of tissues collected day 3 p.i. possessed detectable infectious virus and as such were not included in analyses presented in the main text.
